# Metabolic profiling of gestational diabetes in obese women during pregnancy

**DOI:** 10.1007/s00125-017-4380-6

**Published:** 2017-08-01

**Authors:** Sara L. White, Dharmintra Pasupathy, Naveed Sattar, Scott M. Nelson, Debbie A. Lawlor, Annette L. Briley, Paul T. Seed, Paul Welsh, Lucilla Poston, on behalf of the UPBEAT Consortium

**Affiliations:** 1 Division of Women’s Health, King’s College London, 10th floor North Wing, St Thomas’ Hospital, Westminster Bridge Road, London, SE1 7EH UK; 2 Institute of Cardiovascular and Medical Sciences, University of Glasgow, Glasgow, UK; 3 School of Medicine, University of Glasgow, Glasgow, UK; 4 MRC Integrative Epidemiology Unit at the University of Bristol, University of Bristol, Bristol, UK; 5 School of Social and Community Medicine, University of Bristol, Bristol, UK

**Keywords:** Biomarkers, Gestational diabetes, Lipids, Obesity, Pregnancy, Targeted metabolome

## Abstract

**Aims/hypothesis:**

Antenatal obesity and associated gestational diabetes (GDM) are increasing worldwide. While pre-existing insulin resistance is implicated in GDM in obese women, the responsible metabolic pathways remain poorly described. Our aim was to compare metabolic profiles in blood of obese pregnant women with and without GDM 10 weeks prior to and at the time of diagnosis by OGTT.

**Methods:**

We investigated 646 women, of whom 198 developed GDM, in this prospective cohort study, a secondary analysis of UK Pregnancies Better Eating and Activity Trial (UPBEAT), a multicentre randomised controlled trial of a complex lifestyle intervention in obese pregnant women. Multivariate regression analyses adjusted for multiple testing, and accounting for appropriate confounders including study intervention, were performed to compare obese women with GDM with obese non-GDM women. We measured 163 analytes in serum, plasma or whole blood, including 147 from a targeted NMR metabolome, at time point 1 (mean gestational age 17 weeks 0 days) and time point 2 (mean gestational age 27 weeks 5 days, at time of OGTT) and compared them between groups.

**Results:**

Multiple significant differences were observed in women who developed GDM compared with women without GDM (false discovery rate corrected *p* values <0.05). Most were evident prior to diagnosis. Women with GDM demonstrated raised lipids and lipoprotein constituents in VLDL subclasses, greater triacylglycerol enrichment across lipoprotein particles, higher branched-chain and aromatic amino acids and different fatty acid, ketone body, adipokine, liver and inflammatory marker profiles compared with those without GDM.

**Conclusions/interpretation:**

Among obese pregnant women, differences in metabolic profile, including exaggerated dyslipidaemia, are evident at least 10 weeks prior to a diagnosis of GDM in the late second trimester.

**Electronic supplementary material:**

The online version of this article (doi:10.1007/s00125-017-4380-6) contains peer-reviewed but unedited supplementary material, which is available to authorised users.

## Introduction

Pregnancy is associated with profound changes in metabolism, which facilitate the growth of a healthy fetus and prepare the mother and infant for the energy requirements in the postpartum period. After an initial anabolic stage, a physiologically beneficial increase in insulin resistance enhances fetal availability of metabolic substrates [1]. In contrast, pre-pregnancy insulin resistance, as often observed in obese women, has been implicated in greater risk of gestational diabetes (GDM) and associated fetal adversity [2]. While the metabolic response to pregnancy is recognised to be different in this increasingly prevalent subgroup of the antenatal population [1], the pathways to GDM in obese women remain poorly described. This is of importance as less than one-third of obese women develop GDM.

Insight into the metabolic adaptations to pregnancy and the pathophysiology of complications is facilitated by metabolomics technology, which provides reproducible analytical data. Two recent metabolomic studies of normal pregnancy have described widespread metabolic perturbations that extend beyond the traditional boundaries of insulin resistance to encompass pathways including amino acids, lipoproteins and inflammatory markers [3, 4]. GDM has also been the focus of some recent metabolomic studies, although to date all have included participants from across the maternal weight spectrum, which is itself known to affect the maternal metabolome [5–9]. None has addressed the metabolome in obese women prior to and at the time of GDM diagnosis.

As no distinction is currently made in clinical practice between obese women of lower and higher GDM risk, we recently developed a prediction tool for GDM in obese women. We found that an algorithm including clinical factors and some conventionally measured biomarkers analysed early in the second trimester of pregnancy performed well, although the addition of more complicated metabolomic measures did not augment the performance of the tool [10].

The aim of the present study was to identify differences in metabolites associated with GDM at two time points in gestation in this cohort of obese women. We report on the early second trimester metabolites, formerly not evaluated in detail, as well as those measured at the time of diagnostic OGTT in the late second trimester.

## Methods

### Study design

This prospective cohort study was a secondary analysis using data from the UK Pregnancies Better Eating and Activity Trial (UPBEAT; isrctn.org registration number 89971375). UPBEAT was a multicentre RCT of a complex dietary and physical activity intervention designed to prevent GDM in obese women and reduce the incidence of large-for-gestational-age (LGA) infants [11]. Women with a pre-pregnancy diagnosis of diabetes, essential hypertension, renal disease, systemic lupus erythematosus, antiphospholipid syndrome, sickle-cell disease, thalassaemia, coeliac disease, thyroid disease or current psychosis and those currently prescribed metformin were excluded. The cohort comprised 1555 women recruited between 2009 and 2014; women were >16 years of age, had a BMI ≥30 kg/m^2^ and a singleton pregnancy, and were randomised between 15 weeks 0 days’ and 18 weeks 6 days’ gestation (15^+0^ and 18^+6^) to either the behavioural intervention superimposed on standard antenatal care or standard antenatal care. As the primary outcomes (GDM and LGA infants) did not differ between intervention and control arms, the trial was treated as a cohort for the purposes of this study.

All aspects of the trial, including the analyses in the present study, were approved by the National Health Service Research Ethics Committee (UK Integrated Research Application System; reference 09/H0802/5) and all participants, including women aged 16 and 17 using Fraser guidelines, provided informed written consent [11].

### Participants

This was a complete case analysis including women who had undertaken a diagnostic OGTT, with blood samples at trial entry and at the time of GDM testing, and with complete analyte data at both time points (*n* = 646).

### Procedures

Sociodemographic, clinical and anthropometric information and non-fasting (random) blood samples were provided at time point 1 (15^+0^ to 18^+6^ weeks’ gestation). The trial protocol required inclusion of OGTTs carried out at 27^+0^ to 28^+6^ weeks’ but for this study a clinically pragmatic approach was adopted with OGTTs at 23^+2^ to 30^+0^ weeks’ (mean 27^+5^) included. Diagnosis of GDM was according to International Association of Diabetes and Pregnancy Study Groups (IADPSG) criteria (fasting glucose ≥5.1 mmol/l, 1 h ≥10.0 mmol/l and 2 h ≥8.5 mmol/l in response to a 75 g oral glucose load) [12]. At OGTT (time point 2), an additional sample used for the analyses described below was obtained at the time of the first fasting blood test (fasting ≥10 h). Blood was kept on ice, processed within 2 h and stored at −80°C (whole blood, serum and plasma). Analyses were undertaken by laboratory technicians blinded to participant data.

### Metabolic profiling

A total of 163 analytes were evaluated in plasma, serum or whole blood using a combination of an NMR metabolome and conventional laboratory assays at both time points. A high-throughput NMR metabolomic platform (serum) targeted to multiple pathways with relevance to insulin resistance was employed (http://computationalmedicine.fi/, accessed 10 January 2017) and analyses were carried out in two batches. This NMR metabolite profile has been used for many large-scale epidemiological studies [4, 13–17] and the methodology has been described previously. There are no discernible batch effects [18]. The platform accurately quantifies numerous lipid measures; lipoprotein particles include VLDL subdivided into six subclasses (extremely large, very large, large, medium, small, very small), IDL, LDL subdivided into three subclasses (large, medium, small) and HDL subdivided into four subclasses (very large, large, medium, small). The platform also elucidates the constituents within each lipoprotein particle type (triacylglycerol, total cholesterol, non-esterified cholesterol and cholesteryl ester levels and phospholipid concentrations). Fatty acids, amino acids, glycolysis-related metabolites, ketone bodies and inflammatory markers are also measured. Sixteen analytes, measured using conventional laboratory platforms (ESM Table 1), were selected on the basis of hypothesised/established association with type 2 diabetes, GDM or insulin resistance [19]. These were markers of glucose homeostasis (HbA_1c_, fructosamine, insulin, C-peptide), liver-associated markers (alanine aminotransferase [ALT], aspartate aminotransferase [AST], γ-glutamyl transferase [gGT], sex hormone binding globulin [SHBG]), adipokines (adiponectin, leptin), inflammatory and endothelial markers (high-sensitivity C-reactive protein [hs-CRP], IL-6, tissue plasminogen activator [tPA] antigen and ferritin), vitamin D and human placental lactogen (hPL).

All analytes were evaluated at both time points, except vitamin D, hPL and HbA_1c_ (time point 1) and insulin resistance indices (time point 2: HOMA2-IR, updated HOMA of insulin resistance; HOMA2-%S, updated HOMA of insulin sensitivity; HOMA2-%B, updated HOMA of steady-state beta cell function [20]). Glucose measurements as part of the OGTT were not included in the analysis at time point 2 as these are integral to the diagnosis of GDM.

### Statistical analysis

Analytes were checked for normality; those with non-parametric distribution were log-transformed. Measures were checked for variation for gestational age at sampling and transformed into corrected centiles where required. Demographic characteristics were compared between groups using Student’s *t* test or Mann–Whitney tests for continuous data and *χ* ^2^ tests for categorical data as appropriate. Analyte data at time points 1 and 2 were compared between women who developed GDM (GDM women) and those who did not (non-GDM women). Associations with GDM status were undertaken using univariate and multivariate regression analyses. SD difference between GDM and non-GDM women is reported to enable comparison across multiple measures, originally recorded in differing units. An a priori decision based on known associations was used to identify confounders for multivariate analyses; these were BMI, parity, ethnicity and age. Intervention allocation was additionally included in the model at time point 2 to adjust for any intervention effect on the analytes. A false discovery rate (FDR) approach [21] was employed to reduce the probability of false-positive findings and minimise the effects of multiple testing. Statistical significance was assumed if FDR-corrected *p* values fell below 0.05.

Sensitivity analyses were performed, removing outliers (measures outside four SDs) and restricting measurements at both time points to shorter gestational windows; 15^+0^ to 17^+6^ weeks’ and 26^+0^ to 28^+6^ weeks’. In addition, a sensitivity analysis comparing available data in the whole cohort to complete case data was undertaken.

Statistical analyses were carried out using Stata software, version 14.2 (StataCorp, College Station, TX, USA).

## Results

Of the 1555 women in the UPBEAT trial, 646 who provided blood samples and for whom complete analyte data were available were included (median BMI 35.2 kg/m^2^). Participant characteristics are given in Table 1. Women who developed GDM (*n* = 198) had higher BMI and systolic blood pressure and were older than those who did not develop GDM.

**Table 1:** Characteristics of GDM women vs non-GDM women

Characteristic	No GDM (*n* = 448)	GDM (*n* = 198)	*p* value^a^
Ethnicity			0.35
African	59 (13.2)	38 (19.2)	
Afro-Caribbean	27 (6.0)	14 (7.1)	
South Asian	33 (7.4)	14 (7.1)	
European	290 (64.7)	116 (58.6)	
Other	39 (8.7)	16 (8.1)	
Parity			0.85
Nulliparous	193 (43.1)	89 (44.9)	
Multiparous	255 (56.9)	109 (55.1)	
Current smoking status			0.53
Non-smoker	420 (93.8)	183 (92.4)	
Smoker	28 (6.3)	15 (7.6)	
Age, years	30.5 ± 5.6	31.5 ± 4.6	0.027
Height, cm	164.2 ± 6.6	163.6 ± 7.1	0.29
Weight, kg	95.5 (87.1–105.8)	96.6 (88.9–107.0)	0.17
BMI, kg/m^2^	34.7 (32.6–38.5)	36.1 (33.0–39.4)	<0.001
Systolic BP, mmHg^b^	116.4 ± 10.8	120.6 ± 10.7	<0.001
GA at time point 1, weeks	16.9 ± 1.1	17.0 ± 1.0	0.47
GA at time point 2, weeks	27.7 ± 0.7	27.8 ± 0.6	0.48
Randomisation			0.63
Control	226 (50.4)	104 (52.5)	
Intervention	222 (49.6)	94 (47.5)	

Data are shown as number (%), mean ± SD or median (interquartile range)

^a^ *p* value from *χ* ^2^, Student’s *t* test or Mann–Whitney test as appropriate

^b^Missing data: systolic BP for 12 individuals

GA, gestational age

**Fig. 1:**
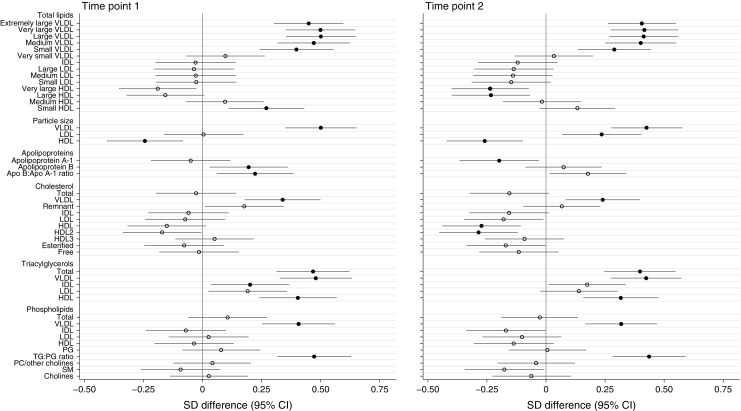
Differences in lipoprotein particle groups between GDM and non-GDM women at time points 1 and 2. Total lipids in all lipoprotein subclasses, particle size, apolipoproteins and total lipoprotein constituents were measured at time points 1 and 2. Data points show the SD difference between GDM and non-GDM women prior to diagnosis of GDM (time point 1) and at the time of OGTT (time point 2). Positive associations with GDM are shown to the right, negative associations are shown to the left. Closed black circles represent FDR-corrected *p* values of <0.05. Free cholesterol, non-esterified cholesterol; PC, phosphatidylcholines; PG, phosphoglycerides; SM, sphingomyelins; TG:PG, triacylglycerol:phosphoglyceride

**Fig. 2:**
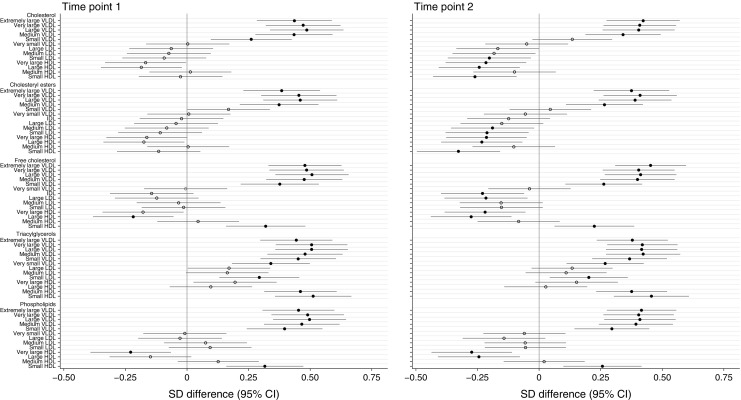
Differences in lipoprotein subclass constituents between GDM and non-GDM women at time points 1 and 2. Lipoprotein subclass constituent contents were measured at time points 1 and 2. Data points show the SD difference between GDM and non-GDM women prior to diagnosis of GDM (time point 1) and at the time of OGTT (time point 2). Positive associations with GDM are shown to the right, negative associations are shown to the left. Closed black circles represent FDR-corrected *p* values of <0.05. Free cholesterol, non-esterified cholesterol

**Fig. 3:**
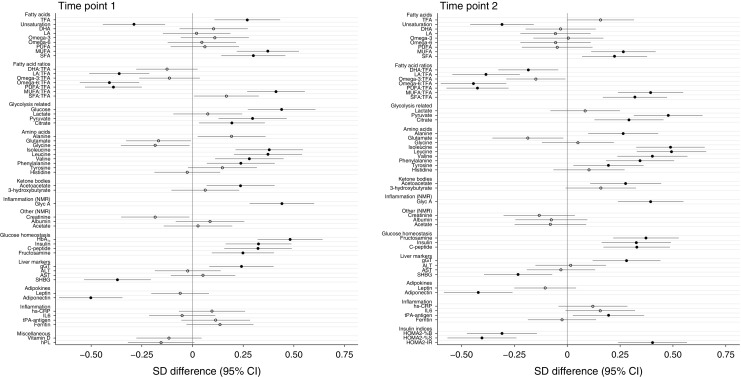
Differences in fatty acids, amino acids, glycaemic and other markers between GDM and non-GDM women at time points 1 and 2. Fatty acids, glycolysis-related metabolites, amino acids, ketone bodies and inflammatory and other markers were measured at time points 1 and 2. Data points show the SD difference between GDM and non-GDM women prior to diagnosis of GDM (time point 1) and at the time of  OGTT (time point 2). Positive associations with GDM are shown to the right, negative associations are shown to the left. Closed black circles represent FDR-corrected *p* values of <0.05. DHA, docosahexaenoic acid, 22:6; LA, linoleic acid, 18:2; MUFA monounsaturated fatty acids, 16:1, 18:1; PUFA polyunsaturated fatty acids; SFA Saturated fatty acids; TFA, total fatty acids

Univariate analysis identified numerous differences between obese GDM and non-GDM women. After adjustment for potential confounders (BMI, ethnicity, parity, age and intervention allocation at time point 2), a similar magnitude of association persisted for most analytes (ESM Tables 2, 3). Association of analytes with GDM, illustrated using difference in SD between GDM and non-GDM women, for the two time points following multivariate analysis are shown in Figs 1, 2, 3. Figure 1 details total lipids in all subclasses, particle size, apolipoproteins and lipoprotein constituents (cholesterol, triacylglycerols and phospholipids) in major lipoprotein groups. Figure 2 gives additional information about constituents in lipoprotein subclasses. Figure 3 details fatty acids, glycolysis-related metabolites, amino acids, ketone bodies and other analytes. Concentrations of these analytes at time points 1 and 2 are shown in ESM Table 4 and ESM Table 5, respectively.

### Lipoproteins

Both before diagnosis and at the time of diagnosis, GDM women (compared with non-GDM women) had higher total lipids in all VLDL subclasses apart from very small (extremely large, very large, large, medium and small) (Fig. 1). Analysis of constituents demonstrated that this was attributable to greater total cholesterol in VLDL (comprising non-esterified cholesterol and cholesteryl esters) in all but the small VLDL subparticles (non-esterified cholesterol alone), together with a higher triacylglycerol concentration in all VLDL subclasses at both time points. VLDL phospholipid content followed a similar pattern: higher in all VLDL subclasses except very small VLDL (Fig. 2).

Prior to diagnosis, total lipids in small HDL were greater in GDM women, a consequence of higher concentrations of non-esterified cholesterol, triacylglycerols and phospholipids. Non-esterified cholesterol concentration was lower in the large HDL subclass and phospholipid concentrations were lower in the very large HDL subclass. As well as VLDL, GDM was positively associated with triacylglycerols in IDL, small LDL and medium and small HDL. Similar differences between GDM and non-GDM women were evident at time point 2, the only additional differences being in cholesterol content of some IDL, LDL and HDL subclasses (Figs 1, 2).

GDM was positively associated with apolipoprotein B and B/A1 ratio at time point 1 but a negative association with apolipoprotein A1 was evident only at the time of diagnosis.

VLDL particles were bigger and HDL smaller in GDM women at both time points. LDL particles were larger at time point 2 (Fig. 1).

### Fatty acids

In GDM compared with non-GDM women, total fatty acids were higher at time point 1 and were marginally increased at the time of diagnosis (Fig. 3). Unsaturation was lower and monounsaturated fatty acid and saturated fatty acid concentrations were greater at both time points. When expressed as proportions of total fatty acids, polyunsaturated fatty acids (linoleic acid, omega-6) were reduced, whereas monounsaturated fatty acids were increased. At time point 2, a decreased proportion of docosahexaenoic acid and increased proportion of saturated fatty acids both reached significance in GDM women.

### Glucose homeostasis

At time point 1, glucose and HbA_1c_ were higher in GDM women and, at both time points, fructosamine, C-peptide and insulin were raised (Fig. 3). Glycolysis intermediate pyruvate and tricarboxylic acid (TCA) cycle intermediate citrate concentrations were also higher but lactate was not different between groups at either time. Insulin indices as assessed by HOMA scores indicated marked reduction in beta cell function and insulin sensitivity as well as increased insulin resistance in GDM women at the time of diagnosis.

### Amino acids and ketone bodies

Branched-chain amino acids (BCAA) valine, leucine and isoleucine were higher in GDM women than in non-GDM women at time points 1 and 2 (Fig. 3). Of the aromatic amino acids, phenylalanine was raised and tyrosine additionally increased in GDM women at time point 2. Alanine was increased at time point 2 and acetoacetate was increased in GDM women at both time points.

### Liver, adipokines and inflammatory markers

Of the liver markers, gGT was markedly higher in GDM women compared with non-GDM women prior to and at the time of diagnosis, whereas SHBG was lower at both time points (Fig. 3). AST and ALT were not associated with GDM status. Adiponectin concentrations were lower in GDM women at both time points but leptin showed no association. The low-grade inflammatory marker glycoprotein acetyls (GlycA) was increased at both time points whereas tPA-antigen was raised only at time point 2 in GDM compared with non-GDM women.

### Sensitivity analyses

Sensitivity analyses (excluding outliers falling outside 4 SD and using a restricted time window) identified minor differences in very few metabolites (9 at time point 1, 20 at time point 2) when compared with the main analyses (ESM Tables 2, 3).

When we repeated analyses using all available data (mean number of women at time point 1 = 925, mean number at time point 2 = 830), apart from one metabolite the results were the same as those presented in the main analyses (complete case analyses). Data are available on request.

## Discussion

Using an approach combining targeted NMR metabolomics and established metabolic risk factors in blood from pregnant women, we have described novel metabolic profile differences in obese women who develop GDM. There was a marked similarity in differential patterns found pre-diagnosis of GDM and at the time of diagnosis. Of the few previous reports of dysglycaemia in pregnancy in which the serum/plasma metabolome has been described, none has previously addressed GDM confined to obese women and recent reports have focused on moderate hyperglycaemia [8, 9].

As anticipated and in line with the diagnosis of GDM, obese women had raised dysglycaemic markers, including HbA_1c_, fructosamine and insulin, and these were raised before diagnosis. Adiponectin, recently identified as a predictor of GDM in women of heterogeneous BMI [22], was markedly lower at both the pre- and peri-diagnosis time points in the obese GDM women compared with the non-GDM women in this cohort.

In accordance with insulin resistance states in non-pregnant individuals [23], the metabolome in GDM revealed a complex change in the metabolic profile. In addition to dysregulation of glucose metabolism, we have demonstrated that obese GDM women, compared with obese non-GDM women, exhibit exaggerated dyslipidaemic profiles that complement our understanding of the effects of insulin resistance on the lipid metabolism pathways in pregnant women [1, 4].

As normal pregnancy progresses, physiological perturbations to glycaemic and lipid pathways caused by increasing insulin resistance are well documented: early lipogenesis (anabolic phase) followed by lipolysis (catabolic phase) is recognised in normal-weight women [1]. Using the same NMR method as the present study, Wang et al reported first trimester lipoprotein concentrations similar to those seen in non-pregnant women in normal non-obese pregnancies, followed by marked increases in lipoprotein constituents, including triacylglycerols, in the second and third trimesters [4]. In contrast, obese women demonstrate reduced insulin sensitivity from an earlier gestation, particularly with regard to lipid metabolism [1]. Using non-NMR methods, others have reported dyslipidaemia in obese vs normal-weight pregnancy, including raised total and LDL-cholesterol, lower HDL-cholesterol and raised triacylglycerols from the first trimester [24–27]. The exaggerated dyslipidaemia in obese women prior to and at the time of diagnosis of GDM in the present study suggests exaggeration of these metabolic processes, reflecting enhanced insulin resistance in adipose tissue and reduced suppression of lipolysis [28]. Potentially compounded by obesity-related higher levels pre-pregnancy, excess fatty acids are thus available for hepatic triacylglycerol synthesis and secretion as VLDL lipoproteins from early gestation. Indeed, all VLDL subclasses were richer in triacylglycerols in GDM compared with non-GDM women, and triacylglycerols were higher in IDL, small LDL and medium and small HDL subclasses, building on reports of triacylglycerol enrichment of VLDL, LDL and HDL in normal pregnancy [29]. The increased concentrations of triacylglycerol-rich lipoproteins may also result from decreased adipose tissue lipoprotein lipase activity, secondary to greater insulin resistance, leading to reduced clearance of triacylglycerol-rich lipoproteins [29]. Cholesteryl ester transferase protein (CETP), the activity of which is enhanced in insulin resistance, likely contributes to the triacylglycerol enrichment of the smaller lipoprotein particles [30]. Furthermore, as observed particularly in later pregnancy samples, the role of CETP is corroborated by increased concentrations of cholesteryl esters in VLDL and reduced quantities in LDL and HDL subclasses [30].

Among the HDL subclasses, the smallest had a different profile from the larger particles, being increased in GDM women. While characterisation of size distribution is divergent between methodological platforms, others using the same NMR platform in a cohort of 9399 Finnish men have reported an abundance of small HDL particles and reduced amounts of larger HDL particles in association with glucose intolerance [23].

Although phospholipid enrichment followed a similar pattern to triacylglycerols, interpretation is limited without further characterisation of these complex lipid subgroups.

There were few consistent changes in either LDL or IDL constituent concentrations in GDM compared with non-GDM women. Contrary to a previous observation in weight-heterogeneous women [31], a relationship between GDM and reduced-sized LDL particles was not observed; indeed, at the time of diagnosis, obese women with GDM exhibited larger LDL particles.

Despite the difference in fasting state between the two points of measurement, similar patterns in fatty acid differences between GDM and non-GDM women were evident. The fatty acid profile in GDM obese women was similar to that seen in a previous study of dysglycaemia in Finnish men using this platform [32]: a predominance of saturated and monounsaturated fatty acids and lower concentrations of polyunsaturated fatty acids in GDM vs non-GDM women. This may reflect the unhealthy dietary pattern termed ‘processed foods’ that we recently reported amongst women who developed GDM in the same cohort [33].

Beyond recognised markers of dysglycaemia (e.g. HbA_1c_ and fructosamine) obese GDM women demonstrated metabolic patterns consistent with perturbed energy pathways and increased fuel availability. These included elevated pyruvate and alanine (both substrates of carbohydrate metabolism), raised acetoacetate (likely secondary to unregulated fatty acid oxidation and/or increased metabolism of BCAA) and higher citrate (an early intermediate of the TCA cycle). The observed increase in alanine concurs with previous metabolomic studies of mild hyperglycaemia in pregnant women of heterogeneous BMI from the Hyperglycemia and Adverse Pregnancy Outcome (HAPO) cohort [8, 9].

As described in weight-heterogeneous GDM women [34] and in recent studies [35] including HAPO [8, 9], BCAA were higher in GDM women, implicating a predominant influence of insulin resistance as opposed to obesity. Whether elevation of BCAA reflects cause or effect is unclear; the mechanism may relate to reduced branch-chain α-ketoacid dehydrogenase activity as implicated in insulin resistance in non-pregnant states, although multiple pathological processes may be involved [36–38]. Consistent with some reports, higher levels of aromatic amino acids were associated with GDM–phenylalanine at both time points and tyrosine later in pregnancy [9, 35]. In contrast, recent studies of the metabolome in normal pregnancy and GDM amongst weight-heterogeneous women demonstrate a complex picture of amino acids across gestation and found no obvious relationship with gestational insulin resistance [4, 35].

gGT was higher in GDM, although other markers of a hepatic process such as fatty liver infiltration (ALT and AST) were not elevated. There is no obvious mechanism, but this could reflect increased oxidative stress, previously linked to GDM [39]. Lower SHBG, associated with insulin resistance [40] and GDM [41], was noted in obese GDM women at both time points, adding to the evidence that low SHBG reflects insulin resistance rather than obesity per se. In contrast to adiponectin, leptin, although reported to be raised in GDM amongst weight-heterogeneous women [42], was not associated with GDM in the obese women. This may reflect habitually high leptin levels in association with obesity, and non-specificity as a GDM marker.

Although inflammatory pathways are commonly implicated in GDM pathogenesis [43], most markers of inflammation measured were not associated with GDM. One notable exception was GlycA, a complex NMR signal of N-acetyl methyl group protons of mobile glycan residues of glycoproteins [44], which was markedly higher in obese GDM women at both time points. Plasma glycoproteins are predominantly acute-phase proteins and GlycA is associated with conditions associated with an inflammatory response, including type 2 diabetes [45]. This novel observation suggests that GlycA might also be a useful marker for GDM.

This study has several strengths and limitations. To our knowledge, this is the first study to assess a range of metabolites associated with GDM in a large cohort of obese pregnant women longitudinally in pregnancy. We are not aware of any study with a similar or larger sample size in obese pregnant women with accurately (universal OGTT) diagnosed GDM and multiple metabolites assessed both in early and mid-pregnancy, and acknowledge that these results should be treated with some caution until replication is possible. This is also the first time that a targeted NMR approach providing detailed lipoprotein and subclass constituent information has addressed GDM. Importantly, BMI was assessed early in pregnancy (at time point 1) prior to fetal growth and with standardised measures in the research setting.

As the trial was designed before recognition of ‘early GDM’, women were not systematically identified as such, or removed, and we acknowledge this as a possible limitation. However, poor correlation between early and later diagnostic testing is recognised [46, 47], exemplified by a recent study of obese women, in which GDM prevalence of ~20% was recorded (OGTT at 24–28 weeks) despite removal of those diagnosed by early OGTT using IADPSG thresholds [48]. This suggests that, despite some overlap, many women diagnosed with GDM at 24–28 weeks’ gestation would not have been classified as GDM early in pregnancy.

A further limitation of the study was that analytes were measured in differing prandial states at the two time points. Samples were collected from non-fasting participants at time point 1, and this could influence some metabolites; however, differential patterns at each time point were not affected. There was also no lean control group comparison.

We recognise that further information could be gained from analysis of the metabolome using mass spectrometry. Particularly interesting, would be non-esterified fatty acids, acylcarnitines and phosphatidylcholine subspecies.

Although highly correlated, NMR metabolite absolute values showed small negative biases compared with conventional platforms for glucose and commonly measured lipids (e.g. total cholesterol) (data not shown). Thus, direct comparison with clinical thresholds is inadvisable.

In summary, this study has increased our knowledge of metabolic pathways in GDM amongst obese pregnant women as assessed by NMR spectroscopy and traditional platforms. It adds to observations in which the metabolome as measured by mass spectrometry was investigated in mildly hyperglycaemic women from the HAPO study (BMI 29.0 kg/m^2^; SD 4.89, at OGTT) [8]. In the present study, we have defined differences in the metabolic profile of GDM specific to obese women. The importance lies in the increasing prevalence of obesity and the current practice of treating all obese women as of equal risk for GDM. The metabolic profiling described has clearly identified that those women who later develop GDM have a similar differential profile earlier in gestation as is evident at the time of GDM diagnosis, when compared with non-GDM women. In addition to classic insulin resistance markers as described recently by others [49], we document a distinct lipid profile characterised by differing lipoprotein subclasses and their constituents. This strongly supports the suggestion that, at least in obese women, the metabolic perturbations of insulin resistance predate GDM diagnosis in the second trimester by many weeks. It follows that diagnosis using glucose thresholds from an OGTT between 24 and 28 weeks fails to identify affected pregnancies in a timely fashion and supports the recent observation that excessive fetal growth precedes the diagnosis of GDM [50]. A diagnostic approach that utilises other metabolic abnormalities rather than or in addition to glucose, a later manifestation of abnormal insulin resistance and function, could potentially improve treatment and outcomes.

This study provides new insight into the metabolic changes associated with GDM in obese women. By demonstrating differences in the metabolic phenotype arising earlier in gestation, encompassing diverse pathways in affected women, this study allows new targets for effective intervention and prevention to be identified. The findings strongly support diagnosis of GDM in obese women earlier in gestation than currently practised, either using the OGTT with new validated thresholds or by means of more biologically relevant risk assessment tools [10].

## Electronic supplementary material

ESM (PDF 1226 kb)

## Abbreviations

ALT: Alanine aminotransferase
AST: Aspartate aminotransferase
BCAA: Branched-chain amino acid
CETP: Cholesteryl ester transferase protein
FDR: False discovery rate
GDM: Gestational diabetes mellitus
gGT: γ-Glutamyl transferase
GlycA: Glycoprotein acetyls
HAPO: Hyperglycemia and Adverse Pregnancy Outcome
HOMA2-%B: Updated HOMA of steady-state beta cell function
HOMA2-IR: Updated HOMA of insulin resistance
HOMA2-%S: Updated HOMA of insulin sensitivity
hPL: Human placental lactogen
hs-CRP: High-sensitivity C-reactive protein
IADPSG: International Association of Diabetes and Pregnancy Study Groups
LGA: Large for gestational age
SHBG: Sex hormone binding globulin
TCA: Tricarboxylic acid
tPA: Tissue plasminogen activator
UPBEAT: UK Pregnancies Better Eating and Activity Trial
