# Supplementary material for: Metabolic profiling of gestational diabetes in obese women during pregnancy
Source: Diabetologia. 2017 Aug 1;60(10):1903–12. doi: 10.1007/s00125-017-4380-6 (PMC6448883; doi:10.1007/s00125-017-4380-6)
Supplement: Supplementary file 1 — (PDF 1226 kb) [file 125_2017_4380_MOESM1_ESM.pdf]

ESM Table 1: Analytical methodologies

Analyte	Sample	Method	Platform	Coefficient of Variation (%) [a]
Insulin	Plasma	Electrochemiluminescence immunoassay	Roche, Cobas e411	< 10.3
C-peptide	Serum	Electrochemiluminescence immunoassay	Roche, Cobas e411	< 6.2
HbA_{1c}	Whole blood	Turbidimetric inhibition immunoassay	Roche, Cobas c311	< 1.4
Fructosamine	Plasma	Colorimetric, nitroblue tetrazolium	Roche, Cobas c311	< 3.4
gGT	Plasma	Enzymatic, colorimetric	Roche, Cobas c311	< 3.9
ALT	Plasma	Enzymatic, spectrophotometric	Roche, Cobas c311	< 3.1
AST	Plasma	Enzymatic, spectrophotometric	Roche, Cobas c311	< 2.1
SHBG	Serum	Electrochemiluminescence immunoassay	Roche, Cobas e411	< 7.9
hs-CRP	Plasma	Particle enhanced immunoturbidimetric	Roche, Cobas c311	< 7.1
Leptin	Plasma	Enzyme-linked immunosorbent assay	R and D Systems	< 2.0
Adiponectin	Plasma	Enzyme-linked immunosorbent assay	R and D Systems	< 6.9
Ferritin	Plasma	Particle enhanced immunoturbidimetric	Roche, Cobas c311	< 1.7
IL-6	Plasma	Enzyme-linked immunosorbent assay	R and D Systems	< 9.8
tPA antigen	Plasma	Enzyme-linked immunosorbent assay	Asserchrom tpa (Stago)	< 5.7
Vitamin D	Serum	Electrochemiluminescence immunoassay	Roche, Cobas e411	< 11.2
hPL	Serum	Enzyme-linked immunosorbent assay	R and D Systems	< 5.0

[a] highest coefficient of variation from either time point 1 or 2 quoted. gGT γ-glutamyl transferase, AST aspartate aminotransferase, ALT alanine aminotransferase, SHBG sex hormone binding globulin, hs-CRP high sensitivity C-reactive protein, IL-6 interleukin-6, tPA-antigen tissue plasminogen activator antigen, hPL human placental lactogen

ESM Table 2: Analyte SD difference between women with and without GDM at time point 1

**Analyte at time point 1**	**Univariate N=646**		**Multivariate N=646**		**Sensitivity N=453**	
	**Mean SD difference** [a]	**95% CI**	**Mean SD difference** [b]	**95% CI**	**Mean SD difference** [c]	**95% CI**
NMR metabolites						
Total lipids						
Total lipids in chylomicrons and extremely large VLDL [d]	**0.35**	(0.19 to 0.52)	**0.45**	(0.30 to 0.60)	**0.46**	(0.29 to 0.64)
Total lipids in very large VLDL [d]	**0.40**	(0.23 to 0.56)	**0.50**	(0.35 to 0.65)	**0.54**	(0.36 to 0.72)
Total lipids in large VLDL [d]	**0.40**	(0.24 to 0.57)	**0.50**	(0.35 to 0.65)	**0.55**	(0.37 to 0.73)
Total lipids in medium VLDL	**0.39**	(0.23 to 0.56)	**0.47**	(0.32 to 0.62)	**0.51**	(0.33 to 0.69)
Total lipids in small VLDL	**0.33**	(0.17 to 0.50)	**0.40**	(0.24 to 0.55)	**0.43**	(0.25 to 0.62)
Total lipids in very small VLDL	0.09	(-0.08 to 0.26)	0.10	(-0.07 to 0.26)	0.11	(-0.09 to 0.30)
Total lipids in IDL	-0.02	(-0.19 to 0.15)	-0.03	(-0.20 to 0.14)	-0.05	(-0.25 to 0.16)
Total lipids in large LDL	-0.03	(-0.20 to 0.14)	-0.04	(-0.21 to 0.13)	-0.06	(-0.26 to 0.14)
Total lipids in medium LDL	-0.02	(-0.19 to 0.15)	-0.03	(-0.20 to 0.14)	-0.05	(-0.25 to 0.15)
Total lipids in small LDL	-0.02	(-0.19 to 0.15)	-0.03	(-0.20 to 0.14)	-0.05	(-0.25 to 0.16)
Total lipids in very large HDL	-0.15	(-0.32 to 0.01)	-0.19	(-0.35 to -0.03)	-0.18	(-0.38 to 0.01)
Total lipids in large HDL	-0.12	(-0.29 to 0.04)	-0.16	(-0.32 to 0.01)	-0.16	(-0.36 to 0.04)
Total lipids in medium HDL	0.08	(-0.08 to 0.25)	0.10	(-0.07 to 0.26)	0.06	(-0.14 to 0.26)
Total lipids in small HDL	**0.26**	(0.09 to 0.42)	**0.27**	(0.11 to 0.43)	0.22	(0.03 to 0.42)
Particle diameter						
Mean diameter for VLDL particles	**0.40**	(0.24 to 0.57)	**0.50**	(0.35 to 0.65)	**0.55**	(0.37 to 0.73)
Mean diameter for LDL particles	0.02	(-0.15 to 0.19)	0.00	(-0.16 to 0.17)	0.04	(-0.16 to 0.24)
Mean diameter for HDL particles	**-0.21**	(-0.37 to -0.04)	**-0.24**	(-0.40 to -0.08)	**-0.23**	(-0.42 to -0.04)
Apolipoproteins						
Apolipoprotein A-1	-0.03	(-0.20 to 0.14)	-0.05	(-0.22 to 0.12)	-0.08	(-0.28 to 0.13)
Apolipoprotein B	0.16	(-0.01 to 0.33)	**0.20**	(0.03 to 0.36)	0.21	(0.01 to 0.40)
Ratio of apolipoprotein B to apolipoprotein A-1	0.18	(0.01 to 0.34)	**0.22**	(0.06 to 0.39)	**0.23**	(0.04 to 0.43)
Cholesterol						
Serum total cholesterol	-0.02	(-0.18 to 0.15)	-0.03	(-0.20 to 0.14)	-0.05	(-0.25 to 0.15)

Total cholesterol in VLDL	**0.29**	(0.12 to 0.46)	**0.34**	(0.18 to 0.50)	**0.36**	(0.18 to 0.55)
Total cholesterol in chylomicrons and extremely large VLDL [d]	**0.35**	(0.18 to 0.51)	**0.44**	(0.28 to 0.59)	**0.46**	(0.27 to 0.64)
Total cholesterol in very large VLDL [d]	**0.38**	(0.21 to 0.54)	**0.47**	(0.32 to 0.62)	**0.51**	(0.33 to 0.69)
Total cholesterol in large VLDL [d]	**0.39**	(0.23 to 0.56)	**0.49**	(0.34 to 0.64)	**0.54**	(0.36 to 0.71)
Total cholesterol in medium VLDL	**0.36**	(0.20 to 0.53)	**0.43**	(0.28 to 0.59)	**0.47**	(0.29 to 0.66)
Total cholesterol in small VLDL	**0.23**	(0.06 to 0.39)	**0.26**	(0.10 to 0.43)	**0.27**	(0.08 to 0.46)
Total cholesterol in very small VLDL	0.02	(-0.15 to 0.19)	0.00	(-0.17 to 0.17)	-0.02	(-0.22 to 0.18)
Remnant cholesterol (non-HDL, non-LDL)	0.16	(-0.01 to 0.32)	0.18	(0.01 to 0.34)	0.18	(-0.01 to 0.38)
Total cholesterol in IDL	-0.04	(-0.21 to 0.12)	-0.06	(-0.23 to 0.11)	-0.09	(-0.29 to 0.12)
Total cholesterol in LDL	-0.06	(-0.23 to 0.11)	-0.07	(-0.24 to 0.10)	-0.10	(-0.30 to 0.10)
Total cholesterol in large LDL	-0.05	(-0.22 to 0.11)	-0.06	(-0.23 to 0.11)	-0.09	(-0.29 to 0.11)
Total cholesterol in medium LDL	-0.06	(-0.23 to 0.10)	-0.07	(-0.24 to 0.10)	-0.10	(-0.30 to 0.10)
Total cholesterol in small LDL	-0.08	(-0.25 to 0.09)	-0.09	(-0.26 to 0.08)	-0.12	(-0.32 to 0.08)
Total cholesterol in HDL	-0.12	(-0.28 to 0.05)	-0.15	(-0.32 to 0.02)	-0.17	(-0.37 to 0.03)
Total cholesterol in HDL2	-0.14	(-0.31 to 0.03)	-0.17	(-0.34 to 0.00)	-0.19	(-0.39 to 0.01)
Total cholesterol in HDL3	0.09	(-0.08 to 0.26)	0.05	(-0.11 to 0.22)	0.05	(-0.15 to 0.24)
Total cholesterol in very large HDL	-0.13	(-0.30 to 0.03)	-0.17	(-0.33 to 0.00)	-0.17	(-0.36 to 0.03)
Total cholesterol in large HDL	-0.15	(-0.32 to 0.02)	-0.19	(-0.35 to -0.02)	-0.18	(-0.38 to 0.01)
Total cholesterol in medium HDL	0.01	(-0.16 to 0.18)	0.01	(-0.15 to 0.18)	-0.02	(-0.22 to 0.17)
Total cholesterol in small HDL	0.02	(-0.15 to 0.18)	-0.03	(-0.20 to 0.14)	-0.09	(-0.29 to 0.11)
Cholesteryl esters						
Esterified cholesterol	-0.08	(-0.24 to 0.09)	-0.08	(-0.25 to 0.09)	-0.11	(-0.31 to 0.09)
Cholesteryl esters in chylomicrons and extremely large VLDL [d]	**0.30**	(0.13 to 0.47)	**0.38**	(0.23 to 0.54)	**0.40**	(0.21 to 0.58)
Cholesteryl esters in very large VLDL [d]	**0.36**	(0.19 to 0.53)	**0.45**	(0.30 to 0.61)	**0.50**	(0.32 to 0.68)
Cholesteryl esters in large VLDL [d]	**0.37**	(0.20 to 0.53)	**0.46**	(0.31 to 0.61)	**0.51**	(0.34 to 0.69)
Cholesteryl esters in medium VLDL	**0.31**	(0.15 to 0.48)	**0.37**	(0.21 to 0.53)	**0.41**	(0.22 to 0.59)
Cholesteryl esters in small VLDL	0.15	(-0.02 to 0.32)	0.17	(0.00 to 0.34)	0.16	(-0.04 to 0.35)
Cholesteryl esters in very small VLDL	0.03	(-0.14 to 0.20)	0.01	(-0.16 to 0.18)	-0.02	(-0.22 to 0.18)
Cholesteryl esters in IDL	-0.01	(-0.18 to 0.16)	-0.02	(-0.19 to 0.15)	-0.05	(-0.25 to 0.16)
Cholesteryl esters in large LDL	-0.04	(-0.21 to 0.13)	-0.04	(-0.21 to 0.13)	-0.07	(-0.27 to 0.13)
Cholesteryl esters in medium LDL	-0.07	(-0.24 to 0.09)	-0.08	(-0.25 to 0.09)	-0.11	(-0.31 to 0.09)
Cholesteryl esters in small LDL	-0.10	(-0.26 to 0.07)	-0.11	(-0.28 to 0.06)	-0.14	(-0.34 to 0.06)
Cholesteryl esters in very large HDL	-0.13	(-0.29 to 0.04)	-0.16	(-0.33 to 0.00)	-0.17	(-0.36 to 0.03)

Cholesteryl esters in large HDL	-0.14	(-0.31	to	0.03)	-0.17	(-0.34	to	-0.01)	-0.17	(-0.37	to	0.02)
Cholesteryl esters in medium HDL	0.00	(-0.17	to	0.17)	0.00	(-0.16	to	0.17)	-0.03	(-0.23	to	0.17)
Cholesteryl esters in small HDL	-0.06	(-0.23	to	0.10)	-0.11	(-0.28	to	0.05)	-0.18	(-0.38	to	0.02)
Non-esterified cholesterol												
Non-esterified cholesterol	-0.02	(-0.19	to	0.15)	-0.01	(-0.18	to	0.15)	-0.01	(-0.21	to	0.19)
Non-esterified cholesterol in chylomicrons and extremely large VLDL [d]	**0.38**	(0.22	to	0.55)	**0.48**	(0.33	to	0.63)	**0.50**	(0.32	to	0.68)
Non-esterified cholesterol in very large VLDL [d]	**0.39**	(0.23	to	0.56)	**0.49**	(0.33	to	0.64)	**0.51**	(0.33	to	0.69)
Non-esterified cholesterol in large VLDL [d]	**0.41**	(0.25	to	0.58)	**0.51**	(0.36	to	0.66)	**0.55**	(0.38	to	0.73)
Non-esterified cholesterol in medium VLDL	**0.40**	(0.23	to	0.56)	**0.48**	(0.32	to	0.63)	**0.52**	(0.34	to	0.70)
Non-esterified cholesterol in small VLDL	**0.32**	(0.15	to	0.49)	**0.38**	(0.22	to	0.54)	**0.41**	(0.23	to	0.60)
Non-esterified cholesterol in very small VLDL	0.00	(-0.17	to	0.17)	-0.01	(-0.17	to	0.16)	0.00	(-0.20	to	0.19)
Non-esterified cholesterol in IDL	-0.12	(-0.29	to	0.05)	-0.14	(-0.31	to	0.03)	-0.17	(-0.38	to	0.03)
Non-esterified cholesterol in large LDL	-0.10	(-0.27	to	0.07)	-0.12	(-0.29	to	0.05)	-0.15	(-0.36	to	0.05)
Non-esterified cholesterol in medium LDL	-0.02	(-0.19	to	0.15)	-0.03	(-0.20	to	0.14)	-0.06	(-0.26	to	0.14)
Non-esterified cholesterol in small LDL	0.00	(-0.17	to	0.17)	-0.01	(-0.18	to	0.16)	-0.03	(-0.24	to	0.17)
Non-esterified cholesterol in very large HDL	-0.14	(-0.31	to	0.02)	-0.18	(-0.34	to	-0.01)	-0.17	(-0.37	to	0.03)
Non-esterified cholesterol in large HDL	-0.18	(-0.35	to	-0.01)	**-0.22**	(-0.38	to	-0.05)	-0.22	(-0.41	to	-0.02)
Non-esterified cholesterol in medium HDL	0.04	(-0.12	to	0.21)	0.05	(-0.12	to	0.21)	0.01	(-0.19	to	0.21)
Non-esterified cholesterol in small HDL	**0.30**	(0.13	to	0.46)	**0.32**	(0.16	to	0.48)	**0.29**	(0.10	to	0.48)
Triacylglycerols												
Serum total triacylglycerols	**0.39**	(0.23	to	0.56)	**0.47**	(0.31	to	0.62)	**0.52**	(0.34	to	0.70)
Triacylglycerols in VLDL	**0.40**	(0.23	to	0.56)	**0.48**	(0.33	to	0.63)	**0.53**	(0.34	to	0.71)
Triacylglycerols in chylomicrons and extremely large VLDL [d]	**0.35**	(0.18	to	0.51)	**0.44**	(0.30	to	0.59)	**0.46**	(0.28	to	0.63)
Triacylglycerols in very large VLDL [d]	**0.40**	(0.24	to	0.57)	**0.51**	(0.36	to	0.65)	**0.55**	(0.38	to	0.73)
Triacylglycerols in large VLDL [d]	**0.41**	(0.24	to	0.57)	**0.51**	(0.36	to	0.65)	**0.56**	(0.38	to	0.73)
Triacylglycerols in medium VLDL	**0.40**	(0.23	to	0.56)	**0.48**	(0.33	to	0.63)	**0.52**	(0.34	to	0.70)
Triacylglycerols in small VLDL	**0.37**	(0.21	to	0.54)	**0.45**	(0.30	to	0.60)	**0.50**	(0.32	to	0.68)
Triacylglycerols in very small VLDL	**0.28**	(0.12	to	0.45)	**0.34**	(0.18	to	0.50)	**0.40**	(0.21	to	0.58)
Triacylglycerols in IDL	0.18	(0.01	to	0.35)	**0.20**	(0.04	to	0.37)	**0.26**	(0.06	to	0.45)
Triacylglycerols in LDL	0.18	(0.01	to	0.34)	0.19	(0.02	to	0.36)	**0.24**	(0.05	to	0.44)
Triacylglycerols in large LDL	0.16	(-0.01	to	0.33)	0.17	(0.00	to	0.34)	0.22	(0.02	to	0.42)

Triacylglycerols in medium LDL	0.15	(-0.02	to	0.32)	0.16	(-0.00	to	0.33)	0.21	(0.01	to	0.41)
Triacylglycerols in small LDL	**0.25**	(0.08	to	0.42)	**0.29**	(0.13	to	0.46)	**0.35**	(0.16	to	0.54)
Triacylglycerols in HDL	**0.36**	(0.20	to	0.53)	**0.40**	(0.24	to	0.57)	**0.46**	(0.27	to	0.66)
Triacylglycerols in very large HDL	0.18	(0.02	to	0.35)	0.19	(0.03	to	0.36)	0.23	(0.03	to	0.43)
Triacylglycerols in large HDL	0.11	(-0.06	to	0.28)	0.10	(-0.07	to	0.26)	0.13	(-0.07	to	0.32)
Triacylglycerols in medium HDL	**0.39**	(0.22	to	0.55)	**0.46**	(0.31	to	0.61)	**0.48**	(0.30	to	0.65)
Triacylglycerols in small HDL	**0.45**	(0.29	to	0.62)	**0.51**	(0.36	to	0.67)	**0.57**	(0.39	to	0.75)
Phospholipids												
Total phospholipids	0.10	(-0.07	to	0.26)	0.11	(-0.06	to	0.27)	0.10	(-0.10	to	0.30)
Phospholipids in VLDL	**0.34**	(0.17	to	0.50)	**0.41**	(0.25	to	0.56)	**0.44**	(0.26	to	0.62)
Phospholipids in chylomicrons and extremely large VLDL [d]	**0.36**	(0.19	to	0.52)	**0.45**	(0.31	to	0.60)	**0.46**	(0.29	to	0.64)
Phospholipids in very large VLDL [d]	**0.39**	(0.23	to	0.56)	**0.49**	(0.34	to	0.64)	**0.52**	(0.34	to	0.69)
Phospholipids in large VLDL [d]	**0.40**	(0.24	to	0.57)	**0.50**	(0.35	to	0.65)	**0.54**	(0.37	to	0.72)
Phospholipids in medium VLDL	**0.39**	(0.22	to	0.55)	**0.47**	(0.31	to	0.62)	**0.51**	(0.33	to	0.69)
Phospholipids in small VLDL	**0.33**	(0.16	to	0.50)	**0.40**	(0.24	to	0.55)	**0.43**	(0.25	to	0.61)
Phospholipids in very small VLDL	-0.01	(-0.18	to	0.16)	-0.01	(-0.18	to	0.16)	-0.01	(-0.21	to	0.19)
Phospholipids in IDL	-0.06	(-0.23	to	0.11)	-0.07	(-0.24	to	0.10)	-0.09	(-0.29	to	0.11)
Phospholipids in LDL	0.03	(-0.14	to	0.19)	0.03	(-0.14	to	0.19)	0.01	(-0.19	to	0.21)
Phospholipids in large LDL	-0.02	(-0.19	to	0.14)	-0.03	(-0.20	to	0.14)	-0.05	(-0.25	to	0.15)
Phospholipids in medium LDL	0.07	(-0.10	to	0.24)	0.08	(-0.09	to	0.24)	0.06	(-0.14	to	0.26)
Phospholipids in small LDL	0.09	(-0.08	to	0.26)	0.09	(-0.07	to	0.26)	0.08	(-0.11	to	0.28)
Phospholipids in very large HDL	-0.19	(-0.36	to	-0.02)	**-0.23**	(-0.39	to	-0.07)	-0.22	(-0.42	to	-0.03)
Phospholipids in HDL	-0.02	(-0.19	to	0.15)	-0.04	(-0.20	to	0.13)	-0.05	(-0.25	to	0.15)
Phospholipids in large HDL	-0.12	(-0.28	to	0.05)	-0.15	(-0.31	to	0.02)	-0.16	(-0.36	to	0.04)
Phospholipids in medium HDL	0.12	(-0.05	to	0.28)	0.13	(-0.04	to	0.29)	0.10	(-0.10	to	0.29)
Phospholipids in small HDL	**0.28**	(0.11	to	0.44)	**0.32**	(0.16	to	0.47)	**0.29**	(0.10	to	0.48)
Total phosphoglycerides	0.05	(-0.12	to	0.22)	0.08	(-0.09	to	0.24)	0.08	(-0.11	to	0.28)
Ratio of triacylglycerols to phosphoglycerides	**0.40**	(0.23	to	0.56)	**0.47**	(0.32	to	0.63)	**0.51**	(0.33	to	0.70)
Phosphatidylcholine and other cholines	0.00	(-0.17	to	0.17)	0.04	(-0.12	to	0.21)	0.02	(-0.17	to	0.22)
Sphingomyelins	-0.07	(-0.24	to	0.10)	-0.09	(-0.26	to	0.07)	-0.14	(-0.34	to	0.06)
Total cholines	0.00	(-0.16	to	0.17)	0.03	(-0.14	to	0.19)	0.02	(-0.18	to	0.22)
Fatty acids												
Total fatty acids	**0.23**	(0.06	to	0.39)	**0.27**	(0.11	to	0.43)	**0.30**	(0.11	to	0.49)

Estimated degree of unsaturation	**-0.21**	(-0.37 to -0.04)	**-0.29**	(-0.44 to -0.14)	**-0.34**	(-0.52 to -0.15)
22:6, docosahexaenoic acid	0.15	(-0.01 to 0.32)	0.10	(-0.06 to 0.27)	0.08	(-0.12 to 0.29)
18:2, linoleic acid	0.02	(-0.15 to 0.18)	0.02	(-0.15 to 0.19)	0.02	(-0.17 to 0.21)
Omega-3 fatty acids	0.15	(-0.02 to 0.31)	0.11	(-0.06 to 0.28)	0.07	(-0.13 to 0.27)
Omega-6 fatty acids	0.05	(-0.12 to 0.22)	0.05	(-0.12 to 0.21)	0.05	(-0.15 to 0.25)
Polyunsaturated fatty acids	0.07	(-0.10 to 0.24)	0.06	(-0.11 to 0.23)	0.06	(-0.14 to 0.26)
Monounsaturated fatty acids; 16:1, 18:1	**0.30**	(0.13 to 0.46)	**0.37**	(0.22 to 0.53)	**0.42**	(0.24 to 0.60)
Saturated fatty acids	**0.24**	(0.08 to 0.41)	**0.30**	(0.14 to 0.46)	**0.34**	(0.15 to 0.53)
Fatty acid ratios						
22:6 docosahexaenoic acid to total fatty acids	-0.03	(-0.19 to 0.14)	-0.13	(-0.28 to 0.03)	-0.19	(-0.38 to -0.01)
18:2 linoleic acid to total fatty acids	**-0.30**	(-0.46 to -0.13)	**-0.36**	(-0.51 to -0.21)	**-0.38**	(-0.56 to -0.21)
Omega-3 fatty acids to total fatty acids	-0.02	(-0.19 to 0.15)	-0.11	(-0.26 to 0.04)	-0.20	(-0.38 to -0.02)
Omega-6 fatty acids to total fatty acids	**-0.32**	(-0.48 to -0.15)	**-0.41**	(-0.56 to -0.26)	**-0.44**	(-0.62 to -0.27)
Polyunsaturated fatty acids to total fatty acids	**-0.28**	(-0.45 to -0.12)	**-0.39**	(-0.53 to -0.25)	**-0.45**	(-0.62 to -0.28)
Monounsaturated fatty acids to total fatty acids	**0.31**	(0.14 to 0.47)	**0.41**	(0.27 to 0.55)	**0.47**	(0.29 to 0.64)
Saturated fatty acids to total fatty acids	0.11	(-0.06 to 0.28)	0.17	(0.01 to 0.33)	0.18	(-0.01 to 0.37)
Glycolysis related						
Glucose	**0.44**	(0.27 to 0.60)	**0.44**	(0.27 to 0.61)	**0.33**	(0.13 to 0.53)
Lactate	0.05	(-0.12 to 0.22)	0.08	(-0.09 to 0.25)	0.08	(-0.12 to 0.29)
Pyruvate	**0.28**	(0.11 to 0.44)	**0.30**	(0.13 to 0.46)	0.23	(0.03 to 0.43)
Citrate	**0.27**	(0.11 to 0.44)	**0.19**	(0.03 to 0.36)	0.18	(-0.02 to 0.37)
Amino acids						
Alanine	0.14	(-0.03 to 0.31)	0.19	(0.03 to 0.36)	0.16	(-0.04 to 0.36)
Glutamine	**-0.25**	(-0.41 to -0.08)	-0.17	(-0.33 to -0.01)	-0.17	(-0.36 to 0.02)
Glycine	**-0.21**	(-0.38 to -0.04)	-0.18	(-0.35 to -0.01)	-0.20	(-0.40 to 0.00)
Branched chain amino acids						
Isoleucine	**0.35**	(0.19 to 0.52)	**0.38**	(0.21 to 0.55)	**0.41**	(0.21 to 0.61)
Leucine	**0.35**	(0.19 to 0.52)	**0.37**	(0.20 to 0.54)	**0.40**	(0.21 to 0.60)
Valine	**0.28**	(0.11 to 0.45)	**0.28**	(0.11 to 0.45)	**0.27**	(0.07 to 0.47)
aromatic amino acids						
Phenylalanine	**0.26**	(0.09 to 0.42)	**0.24**	(0.07 to 0.41)	**0.28**	(0.08 to 0.48)
Tyrosine	0.16	(-0.01 to 0.33)	0.15	(-0.02 to 0.32)	0.22	(0.02 to 0.42)

Histidine	-0.10	(-0.27 to 0.07)	-0.03	(-0.19 to 0.14)	0.08	(-0.11 to 0.28)
Ketone bodies						
Acetoacetate [d]	**0.27**	(0.10 to 0.44)	**0.24**	(0.07 to 0.40)	0.17	(-0.03 to 0.37)
3-hydroxybutyrate [d]	0.14	(-0.03 to 0.30)	0.06	(-0.10 to 0.23)	0.04	(-0.16 to 0.24)
Inflammatory marker (NMR)						
Glycoprotein acetlys, mainly a1-acid glycoprotein	**0.43**	(0.26 to 0.59)	**0.44**	(0.28 to 0.60)	**0.40**	(0.21 to 0.59)
Other (NMR)						
Creatinine	-0.13	(-0.29 to 0.04)	-0.18	(-0.35 to -0.02)	-0.12	(-0.32 to 0.08)
Albumin	0.10	(-0.07 to 0.26)	0.09	(-0.08 to 0.26)	0.09	(-0.11 to 0.29)
Acetate [d]	0.04	(-0.13 to 0.20)	0.03	(-0.14 to 0.20)	0.03	(-0.17 to 0.23)
Conventionally measured analytes						
Glucose homeostasis						
HbA_{1c}	**0.56**	(0.39 to 0.72)	**0.48**	(0.32 to 0.64)	**0.43**	(0.24 to 0.62)
Insulin [d]	**0.36**	(0.20 to 0.53)	**0.33**	(0.16 to 0.49)	**0.31**	(0.12 to 0.51)
C-peptide [d]	**0.34**	(0.17 to 0.50)	**0.32**	(0.16 to 0.49)	**0.30**	(0.10 to 0.50)
Fructosamine	**0.30**	(0.14 to 0.47)	**0.25**	(0.10 to 0.40)	0.21	(0.02 to 0.39)
**Liver markers**						
gGT [d]	**0.31**	(0.14 to 0.48)	**0.24**	(0.08 to 0.40)	**0.28**	(0.10 to 0.47)
ALT [d]	0.03	(-0.14 to 0.20)	-0.02	(-0.19 to 0.14)	0.02	(-0.18 to 0.21)
AST [d]	0.12	(-0.05 to 0.29)	0.05	(-0.11 to 0.21)	0.10	(-0.09 to 0.29)
SHBG	**-0.39**	(-0.55 to -0.22)	**-0.37**	(-0.53 to -0.21)	**-0.43**	(-0.63 to -0.24)
**Adipokines**						
Leptin [d]	0.09	(-0.08 to 0.26)	-0.06	(-0.20 to 0.08)	-0.02	(-0.20 to 0.15)
Adiponectin [d]	**-0.54**	(-0.71 to -0.38)	**-0.50**	(-0.66 to -0.35)	**-0.48**	(-0.67 to -0.29)
**Inflammatory markers**						
hs-CRP [d]	0.14	(-0.03 to 0.31)	0.10	(-0.07 to 0.26)	0.01	(-0.19 to 0.20)
IL-6 [d]	0.02	(-0.15 to 0.19)	-0.05	(-0.21 to 0.11)	-0.06	(-0.25 to 0.13)
tPA-antigen [d]	0.16	(-0.01 to 0.32)	0.11	(-0.05 to 0.28)	0.14	(-0.06 to 0.35)
Ferritin [d]	0.16	(-0.01 to 0.32)	0.14	(-0.03 to 0.30)	0.19	(-0.01 to 0.38)
**Miscellaneous**						
Vitamin D [d]	-0.16	(-0.33 to 0.01)	-0.12	(-0.28 to 0.04)	-0.12	(-0.31 to 0.07)
hPL [e]	**-0.23**	(-0.40 to -0.06)	-0.15	(-0.32 to 0.01)	-0.10	(-0.30 to 0.09)

[a b c] False Discovery Rate (FDR) corrected *p* value <0.05 shown in bold [d] analyte log transformed [e] analyte z score. GDM gestational diabetes mellitus, gGT γ-glutamyl transferase, AST aspartate aminotransferase, ALT alanine aminotransferase, SHBG sex hormone binding globulin, hs-CRP high sensitivity C-reactive protein, IL-6 interleukin-6, tPA-antigen tissue plasminogen activator antigen, hPL human placental lactogen

ESM Table 3: Analyte SD difference between women with and without GDM at time point 2

Analyte at time point 2	Univariate N= 646 Mean SD Difference [a]	95% CI		Multivariate N=646 Mean SD Difference [b]	95% CI		Sensitivity N= 568 Mean SD Difference [c]	95% CI	
NMR metabolites									
Total lipids									
Total lipids in chylomicrons and extremely large VLDL [d]	**0.31**	(0.14 to	0.47)	**0.41**	(0.26 to	0.55)	**0.39**	(0.24 to	0.55)
Total lipids in very large VLDL [d]	**0.32**	(0.15 to	0.48)	**0.42**	(0.27 to	0.56)	**0.40**	(0.24 to	0.55)
Total lipids in large VLDL [d]	**0.32**	(0.15 to	0.48)	**0.41**	(0.27 to	0.56)	**0.39**	(0.23 to	0.54)
Total lipids in medium VLDL	**0.32**	(0.16 to	0.49)	**0.40**	(0.25 to	0.55)	**0.36**	(0.20 to	0.52)
Total lipids in small VLDL	**0.22**	(0.05 to	0.39)	**0.29**	(0.14 to	0.44)	**0.27**	(0.10 to	0.43)
Total lipids in very small VLDL	0.01	(-0.16 to	0.18)	0.03	(-0.13 to	0.20)	0.06	(-0.11 to	0.24)
Total lipids in IDL	-0.13	(-0.29 to	0.04)	-0.12	(-0.29 to	0.05)	-0.06	(-0.24 to	0.12)
Total lipids in large LDL	-0.14	(-0.31 to	0.02)	-0.14	(-0.30 to	0.03)	-0.08	(-0.26 to	0.10)
Total lipids in medium LDL	-0.15	(-0.32 to	0.02)	-0.14	(-0.31 to	0.03)	-0.08	(-0.26 to	0.10)
Total lipids in small LDL	-0.16	(-0.32 to	0.01)	-0.15	(-0.31 to	0.02)	-0.09	(-0.27 to	0.09)
Total lipids in very large HDL	**-0.20**	(-0.37 to	-0.03)	**-0.24**	(-0.40 to	-0.07)	-0.16	(-0.33 to	0.02)
Total lipids in large HDL	**-0.20**	(-0.37 to	-0.03)	**-0.23**	(-0.40 to	-0.07)	-0.16	(-0.34 to	0.02)
Total lipids in medium HDL	-0.03	(-0.20 to	0.14)	-0.02	(-0.18 to	0.15)	-0.01	(-0.18 to	0.17)
Total lipids in small HDL	0.10	(-0.06 to	0.27)	0.13	(-0.03 to	0.29)	0.10	(-0.06 to	0.27)
Particle diameter									
Mean diameter for VLDL particles	**0.33**	(0.17 to	0.50)	**0.43**	(0.28 to	0.58)	**0.39**	(0.23 to	0.55)
Mean diameter for LDL particles	**0.25**	(0.09 to	0.42)	**0.24**	(0.07 to	0.40)	**0.23**	(0.05 to	0.41)
Mean diameter for HDL particles	**-0.22**	(-0.38 to	-0.05)	**-0.26**	(-0.42 to	-0.10)	-0.18	(-0.35 to	-0.01)
Apolipoproteins									

Apolipoprotein A-1	-0.18	(-0.35 to	-0.02)	**-0.20**	(-0.37 to	-0.03)	-0.12	(-0.30 to	0.06)
Apolipoprotein B	0.03	(-0.13 to	0.20)	0.07	(-0.09 to	0.24)	0.10	(-0.07 to	0.27)
Ratio of apolipoprotein B to apolipoprotein A-1	0.13	(-0.04 to	0.30)	0.18	(0.01 to	0.34)	0.16	(-0.01 to	0.34)
Cholesterol									
Serum total cholesterol	-0.16	(-0.33 to	0.01)	-0.15	(-0.32 to	0.01)	-0.09	(-0.26 to	0.09)
Total cholesterol in VLDL	0.18	(0.02 to	0.35)	**0.24**	(0.08 to	0.40)	**0.24**	(0.07 to	0.41)
Total cholesterol in chylomicrons and extremely large VLDL [d]	**0.33**	(0.17 to	0.50)	**0.42**	(0.27 to	0.57)	**0.42**	(0.27 to	0.58)
Total cholesterol in very large VLDL [d]	**0.31**	(0.15 to	0.48)	**0.41**	(0.26 to	0.56)	**0.40**	(0.24 to	0.55)
Total cholesterol in large VLDL [d]	**0.31**	(0.14 to	0.47)	**0.40**	(0.26 to	0.55)	**0.38**	(0.23 to	0.54)
Total cholesterol in medium VLDL	**0.27**	(0.10 to	0.43)	**0.34**	(0.19 to	0.49)	**0.32**	(0.16 to	0.48)
Total cholesterol in small VLDL	0.09	(-0.08 to	0.26)	0.13	(-0.03 to	0.30)	0.15	(-0.03 to	0.32)
Total cholesterol in very small VLDL	-0.05	(-0.22 to	0.11)	-0.05	(-0.22 to	0.12)	0.00	(-0.18 to	0.18)
Remnant cholesterol (non-HDL, non-LDL)	0.03	(-0.14 to	0.20)	0.07	(-0.10 to	0.23)	0.10	(-0.07 to	0.27)
Total cholesterol in IDL	-0.16	(-0.32 to	0.01)	-0.16	(-0.32 to	0.01)	-0.09	(-0.27 to	0.09)
Total cholesterol in LDL	-0.18	(-0.35 to	-0.02)	-0.18	(-0.35 to	-0.01)	-0.12	(-0.30 to	0.06)
Total cholesterol in large LDL	-0.17	(-0.34 to	0.00)	-0.17	(-0.34 to	0.00)	-0.10	(-0.28 to	0.08)
Total cholesterol in medium LDL	-0.19	(-0.35 to	-0.02)	-0.18	(-0.35 to	-0.01)	-0.12	(-0.30 to	0.06)
Total cholesterol in small LDL	**-0.20**	(-0.37 to	-0.04)	**-0.20**	(-0.37 to	-0.03)	-0.14	(-0.32 to	0.04)
Total cholesterol in HDL	**-0.24**	(-0.40 to	-0.07)	**-0.27**	(-0.44 to	-0.11)	-0.20	(-0.37 to	-0.02)
Total cholesterol in HDL2	**-0.25**	(-0.42 to	-0.08)	**-0.29**	(-0.45 to	-0.12)	**-0.21**	(-0.39 to	-0.03)
Total cholesterol in HDL3	-0.07	(-0.24 to	0.10)	-0.09	(-0.26 to	0.08)	-0.01	(-0.19 to	0.17)
Total cholesterol in very large HDL	-0.18	(-0.35 to	-0.01)	**-0.22**	(-0.38 to	-0.05)	-0.14	(-0.31 to	0.04)
Total cholesterol in large HDL	**-0.21**	(-0.37 to	-0.04)	**-0.24**	(-0.41 to	-0.08)	-0.17	(-0.35 to	0.01)
Total cholesterol in medium HDL	-0.10	(-0.27 to	0.07)	-0.10	(-0.27 to	0.07)	-0.08	(-0.26 to	0.09)
Total cholesterol in small HDL	**-0.24**	(-0.40 to	-0.07)	**-0.26**	(-0.43 to	-0.09)	**-0.22**	(-0.40 to	-0.04)
Cholesteryl esters									
Esterified cholesterol	-0.17	(-0.34 to	-0.01)	-0.17	(-0.34 to	0.00)	-0.10	(-0.28 to	0.08)
Cholesteryl esters in chylomicrons and extremely large VLDL [d]	**0.29**	(0.13 to	0.46)	**0.38**	(0.22 to	0.53)	**0.39**	(0.23 to	0.55)

Cholesteryl esters in very large VLDL [d]	**0.31**	(0.15 to 0.48)	**0.41**	(0.26 to 0.56)	**0.39**	(0.23 to 0.55)
Cholesteryl esters in large VLDL [d]	**0.29**	(0.13 to 0.46)	**0.39**	(0.24 to 0.54)	**0.37**	(0.21 to 0.53)
Cholesteryl esters in medium VLDL	**0.20**	(0.03 to 0.37)	**0.27**	(0.11 to 0.42)	**0.26**	(0.10 to 0.43)
Cholesteryl esters in small VLDL	0.02	(-0.15 to 0.18)	0.05	(-0.12 to 0.21)	0.07	(-0.10 to 0.25)
Cholesteryl esters in very small VLDL	-0.05	(-0.22 to 0.11)	-0.06	(-0.22 to 0.11)	0.00	(-0.18 to 0.18)
Cholesteryl esters in IDL	-0.13	(-0.30 to 0.04)	-0.12	(-0.29 to 0.04)	-0.06	(-0.24 to 0.12)
Cholesteryl esters in large LDL	-0.16	(-0.33 to 0.01)	-0.15	(-0.32 to 0.02)	-0.09	(-0.27 to 0.09)
Cholesteryl esters in medium LDL	**-0.19**	(-0.36 to -0.03)	**-0.19**	(-0.36 to -0.02)	-0.13	(-0.31 to 0.05)
Cholesteryl esters in small LDL	**-0.21**	(-0.38 to -0.04)	**-0.21**	(-0.38 to -0.04)	-0.15	(-0.33 to 0.03)
Cholesteryl esters in very large HDL	-0.18	(-0.35 to -0.01)	**-0.21**	(-0.38 to -0.05)	-0.13	(-0.31 to 0.04)
Cholesteryl esters in large HDL	**-0.20**	(-0.36 to -0.03)	**-0.23**	(-0.40 to -0.07)	-0.16	(-0.34 to 0.02)
Cholesteryl esters in medium HDL	-0.10	(-0.27 to 0.06)	-0.10	(-0.27 to 0.06)	-0.09	(-0.27 to 0.09)
Cholesteryl esters in small HDL	**-0.29**	(-0.46 to -0.13)	**-0.33**	(-0.50 to -0.16)	**-0.28**	(-0.46 to -0.10)
Non-esterified cholesterol						
Non-esterified cholesterol	-0.12	(-0.29 to 0.04)	-0.12	(-0.28 to 0.05)	-0.05	(-0.23 to 0.13)
Non-esterified cholesterol in chylomicrons and extremely large VLDL [d]	**0.35**	(0.19 to 0.52)	**0.45**	(0.31 to 0.60)	**0.44**	(0.29 to 0.60)
Non-esterified cholesterol in very large VLDL [d]	**0.31**	(0.14 to 0.47)	**0.40**	(0.26 to 0.55)	**0.40**	(0.24 to 0.55)
Non-esterified cholesterol in large VLDL [d]	**0.32**	(0.15 to 0.48)	**0.41**	(0.27 to 0.56)	**0.39**	(0.24 to 0.55)
Non-esterified cholesterol in medium VLDL	**0.32**	(0.16 to 0.49)	**0.40**	(0.25 to 0.55)	**0.36**	(0.20 to 0.53)
Non-esterified cholesterol in small VLDL	**0.20**	(0.03 to 0.37)	**0.26**	(0.11 to 0.42)	**0.25**	(0.08 to 0.41)
Non-esterified cholesterol in very small VLDL	-0.05	(-0.22 to 0.12)	-0.04	(-0.21 to 0.13)	0.00	(-0.18 to 0.18)
Non-esterified cholesterol in IDL	**-0.22**	(-0.39 to -0.05)	**-0.23**	(-0.40 to -0.06)	-0.16	(-0.34 to 0.03)
Non-esterified cholesterol in large LDL	**-0.21**	(-0.38 to -0.04)	**-0.22**	(-0.38 to -0.05)	-0.14	(-0.33 to 0.04)
Non-esterified cholesterol in medium LDL	-0.16	(-0.32 to 0.01)	-0.15	(-0.32 to 0.01)	-0.09	(-0.27 to 0.09)
Non-esterified cholesterol in small LDL	-0.16	(-0.32 to 0.01)	-0.15	(-0.32 to 0.02)	-0.09	(-0.27 to 0.09)
Non-esterified cholesterol in very large HDL	-0.18	(-0.35 to -0.02)	**-0.22**	(-0.38 to -0.06)	-0.14	(-0.31 to 0.04)
Non-esterified cholesterol in large HDL	**-0.23**	(-0.40 to -0.07)	**-0.28**	(-0.44 to -0.11)	-0.20	(-0.37 to -0.02)

Non-esterified cholesterol in medium HDL	-0.08	(-0.25 to	0.08)	-0.08	(-0.25 to	0.08)	-0.06	(-0.23 to	0.12)
Non-esterified cholesterol in small HDL	**0.20**	(0.03 to	0.36)	**0.22**	(0.06 to	0.39)	0.19	(0.02 to	0.36)
Triacylglycerols									
Serum total triacylglycerols	**0.32**	(0.16 to	0.49)	**0.40**	(0.25 to	0.55)	**0.36**	(0.20 to	0.52)
Triacylglycerols in VLDL	**0.34**	(0.18 to	0.51)	**0.42**	(0.27 to	0.57)	**0.38**	(0.22 to	0.54)
Triacylglycerols in chylomicrons and extremely large VLDL [d]	**0.28**	(0.12 to	0.45)	**0.38**	(0.23 to	0.52)	**0.36**	(0.21 to	0.51)
Triacylglycerols in very large VLDL [d]	**0.32**	(0.15 to	0.48)	**0.42**	(0.28 to	0.56)	**0.40**	(0.24 to	0.55)
Triacylglycerols in large VLDL [d]	**0.32**	(0.15 to	0.48)	**0.42**	(0.27 to	0.56)	**0.39**	(0.23 to	0.55)
Triacylglycerols in medium VLDL	**0.34**	(0.18 to	0.51)	**0.42**	(0.27 to	0.57)	**0.38**	(0.22 to	0.54)
Triacylglycerols in small VLDL	**0.29**	(0.12 to	0.46)	**0.37**	(0.22 to	0.52)	**0.32**	(0.16 to	0.49)
Triacylglycerols in very small VLDL	**0.21**	(0.04 to	0.37)	**0.27**	(0.11 to	0.42)	**0.24**	(0.08 to	0.41)
Triacylglycerols in IDL	0.14	(-0.03 to	0.30)	0.17	(0.01 to	0.34)	0.17	(-0.00 to	0.35)
Triacylglycerols in LDL	0.11	(-0.06 to	0.27)	0.14	(-0.02 to	0.30)	0.15	(-0.02 to	0.33)
Triacylglycerols in large LDL	0.10	(-0.06 to	0.27)	0.13	(-0.03 to	0.30)	0.15	(-0.03 to	0.33)
Triacylglycerols in medium LDL	0.08	(-0.09 to	0.25)	0.11	(-0.05 to	0.27)	0.13	(-0.05 to	0.30)
Triacylglycerols in small LDL	0.15	(-0.02 to	0.31)	**0.20**	(0.04 to	0.36)	**0.20**	(0.03 to	0.37)
Triacylglycerols in HDL	**0.26**	(0.10 to	0.43)	**0.32**	(0.16 to	0.48)	**0.32**	(0.15 to	0.50)
Triacylglycerols in very large HDL	0.13	(-0.04 to	0.29)	0.15	(-0.02 to	0.32)	0.19	(0.01 to	0.37)
Triacylglycerols in large HDL	0.03	(-0.14 to	0.19)	0.03	(-0.14 to	0.20)	0.09	(-0.09 to	0.27)
Triacylglycerols in medium HDL	**0.30**	(0.13 to	0.46)	**0.38**	(0.23 to	0.52)	**0.34**	(0.19 to	0.49)
Triacylglycerols in small HDL	**0.39**	(0.22 to	0.55)	**0.46**	(0.30 to	0.61)	**0.41**	(0.24 to	0.57)
Phospholipids									
Total phospholipids	-0.05	(-0.22 to	0.12)	-0.03	(-0.19 to	0.14)	0.03	(-0.15 to	0.20)
Phospholipids in VLDL	**0.25**	(0.08 to	0.41)	**0.32**	(0.17 to	0.47)	**0.30**	(0.14 to	0.46)
Phospholipids in chylomicrons and extremely large VLDL [d]	**0.32**	(0.15 to	0.48)	**0.42**	(0.28 to	0.56)	**0.40**	(0.25 to	0.55)
Phospholipids in very large VLDL [d]	**0.31**	(0.14 to	0.47)	**0.40**	(0.26 to	0.55)	**0.39**	(0.24 to	0.54)
Phospholipids in large VLDL [d]	**0.31**	(0.15 to	0.48)	**0.41**	(0.26 to	0.55)	**0.39**	(0.23 to	0.54)
Phospholipids in medium VLDL	**0.31**	(0.15 to	0.48)	**0.39**	(0.24 to	0.54)	**0.36**	(0.20 to	0.52)
Phospholipids in small VLDL	**0.22**	(0.06 to	0.39)	**0.30**	(0.14 to	0.45)	**0.27**	(0.11 to	0.43)

Phospholipids in very small VLDL	-0.07	(-0.24 to	0.09)	-0.06	(-0.23 to	0.11)	-0.02	(-0.20 to	0.16)
Phospholipids in IDL	-0.17	(-0.34 to	-0.01)	-0.17	(-0.34 to	0.00)	-0.10	(-0.28 to	0.08)
Phospholipids in LDL	-0.12	(-0.25 to	0.05)	-0.10	(-0.27 to	0.06)	-0.03	(-0.21 to	0.14)
Phospholipids in large LDL	-0.15	(-0.32 to	0.02)	-0.14	(-0.31 to	0.03)	-0.08	(-0.26 to	0.10)
Phospholipids in medium LDL	-0.08	(-0.25 to	0.09)	-0.06	(-0.22 to	0.11)	-0.01	(-0.18 to	0.17)
Phospholipids in small LDL	-0.08	(-0.25 to	0.09)	-0.06	(-0.22 to	0.11)	0.00	(-0.18 to	0.17)
Phospholipids in very large HDL	**-0.23**	(-0.40 to	-0.07)	**-0.27**	(-0.44 to	-0.11)	**-0.19**	(-0.37 to	-0.02)
Phospholipids in HDL	-0.12	(-0.29 to	0.05)	-0.14	(-0.30 to	0.03)	-0.10	(-0.28 to	0.08)
Phospholipids in large HDL	**-0.21**	(-0.38 to	-0.04)	**-0.24**	(-0.41 to	-0.08)	-0.17	(-0.35 to	0.01)
Phospholipids in medium HDL	0.01	(-0.16 to	0.17)	0.02	(-0.14 to	0.19)	0.03	(-0.14 to	0.21)
Phospholipids in small HDL	**0.22**	(0.05 to	0.38)	**0.26**	(0.10 to	0.42)	**0.20**	(0.03 to	0.37)
Total phosphoglycerides	-0.01	(-0.18 to	0.15)	0.01	(-0.16 to	0.17)	0.07	(-0.11 to	0.24)
Ratio of triacylglycerols to phosphoglycerides	**0.37**	(0.20 to	0.53)	**0.44**	(0.28 to	0.59)	**0.38**	(0.22 to	0.55)
Phosphatidylcholine and other cholines	-0.07	(-0.24 to	0.10)	-0.04	(-0.21 to	0.12)	0.02	(-0.15 to	0.20)
Sphingomyelins	-0.16	(-0.33 to	0.01)	-0.18	(-0.34 to	-0.01)	-0.11	(-0.29 to	0.07)
Total cholines	-0.07	(-0.24 to	0.10)	-0.06	(-0.23 to	0.10)	0.01	(-0.17 to	0.19)
Fatty acids									
Total fatty acids	0.11	(-0.06 to	0.28)	0.16	(-0.00 to	0.32)	0.19	(0.02 to	0.36)
Estimated degree of unsaturation	**-0.20**	(-0.37 to	-0.04)	**-0.31**	(-0.46 to	-0.16)	**-0.24**	(-0.40 to	-0.08)
22:6, docosahexaenoic acid	0.03	(-0.13 to	0.20)	-0.03	(-0.20 to	0.13)	0.00	(-0.18 to	0.18)
18:2, linoleic acid	-0.08	(-0.25 to	0.08)	-0.06	(-0.22 to	0.11)	0.01	(-0.16 to	0.19)
Omega-3 fatty acids	0.06	(-0.10 to	0.23)	0.01	(-0.16 to	0.17)	0.05	(-0.13 to	0.23)
Omega-6 fatty acids	-0.07	(-0.24 to	0.09)	-0.06	(-0.22 to	0.11)	0.02	(-0.16 to	0.19)
Polyunsaturated fatty acids	-0.05	(-0.22 to	0.11)	-0.05	(-0.21 to	0.12)	0.02	(-0.16 to	0.20)
Monounsaturated fatty acids; 16:1, 18:1	**0.21**	(0.04 to	0.37)	**0.27**	(0.11 to	0.42)	**0.27**	(0.11 to	0.43)
Saturated fatty acids	0.16	(-0.00 to	0.33)	**0.22**	(0.07 to	0.38)	**0.23**	(0.07 to	0.40)
Ratio of 22:6 docosahexaenoic acid to total fatty acids	-0.06	(-0.22 to	0.11)	**-0.18**	(-0.33 to	-0.04)	**-0.19**	(-0.34 to	-0.04)
Ratio of 18:2 linoleic acid to total fatty acids	**-0.36**	(-0.52 to	-0.19)	**-0.38**	(-0.54 to	-0.22)	**-0.30**	(-0.47 to	-0.13)
Ratio of omega-3 fatty acids to total fatty	-0.02	(-0.19 to	0.15)	-0.15	(-0.29 to	-0.01)	-0.14	(-0.29 to	0.01)

acids									
Ratio of omega-6 fatty acids to total fatty acids	**-0.38**	(-0.55 to	-0.22)	**-0.44**	(-0.60 to	-0.29)	**-0.36**	(-0.53 to	-0.19)
Ratio of polyunsaturated fatty acids to total fatty acids	**-0.33**	(-0.50 to	-0.17)	**-0.42**	(-0.57 to	-0.28)	**-0.35**	(-0.51 to	-0.19)
Ratio of monounsaturated fatty acids to total fatty acids	**0.32**	(0.16 to	0.49)	**0.39**	(0.24 to	0.55)	**0.34**	(0.17 to	0.50)
Ratio of saturated fatty acids to total fatty acids	**0.24**	(0.07 to	0.40)	**0.32**	(0.17 to	0.47)	**0.24**	(0.07 to	0.40)
Glycolysis related									
Lactate	0.07	(-0.10 to	0.23)	0.08	(-0.08 to	0.25)	0.07	(-0.10 to	0.25)
Pyruvate	**0.46**	(0.29 to	0.62)	**0.48**	(0.31 to	0.64)	**0.48**	(0.31 to	0.66)
Citrate	**0.37**	(0.20 to	0.53)	**0.29**	(0.13 to	0.45)	**0.31**	(0.13 to	0.48)
Amino acids									
Alanine	**0.23**	(0.06 to	0.40)	**0.26**	(0.10 to	0.43)	**0.24**	(0.06 to	0.41)
Glutamine	**-0.22**	(-0.38 to	-0.05)	-0.19	(-0.35 to	-0.02)	-0.19	(-0.37 to	-0.01)
Glycine	0.06	(-0.11 to	0.23)	0.05	(-0.12 to	0.22)	0.04	(-0.15 to	0.22)
Branched chain amino acids									
Isoleucine	**0.46**	(0.30 to	0.62)	**0.49**	(0.33 to	0.65)	**0.48**	(0.31 to	0.65)
Leucine	**0.49**	(0.32 to	0.65)	**0.49**	(0.33 to	0.66)	**0.48**	(0.30 to	0.65)
Valine	**0.42**	(0.26 to	0.59)	**0.40**	(0.24 to	0.57)	**0.40**	(0.22 to	0.58)
Aromatic amino acids									
Phenylalanine	**0.38**	(0.22 to	0.55)	**0.34**	(0.18 to	0.51)	**0.35**	(0.18 to	0.52)
Tyrosine	**0.23**	(0.07 to	0.40)	**0.20**	(0.03 to	0.36)	0.18	(-0.00 to	0.35)
Histidine	0.06	(-0.11 to	0.23)	0.10	(-0.07 to	0.27)	0.07	(-0.11 to	0.25)
Ketone bodies									
Acetoacetate [d]	**0.30**	(0.13 to	0.47)	**0.28**	(0.11 to	0.44)	**0.27**	(0.09 to	0.45)
3-hydroxybutyrate [d]	**0.23**	(0.06 to	0.39)	0.16	(-0.01 to	0.33)	0.18	(-0.00 to	0.35)
Inflammatory marker (NMR)									
Glycoprotein acetlys, mainly a1-acid glycoprotein	**0.37**	(0.21 to	0.54)	**0.39**	(0.24 to	0.55)	**0.42**	(0.25 to	0.58)
Other (NMR)									

Creatinine	-0.09	(-0.26 to	0.08)	-0.13	(-0.30 to	0.04)	-0.12	(-0.29 to	0.06)
Albumin	-0.04	(-0.21 to	0.13)	-0.08	(-0.25 to	0.09)	0.00	(-0.18 to	0.18)
Acetate [d]	-0.07	(-0.24 to	0.10)	-0.08	(-0.25 to	0.09)	-0.07	(-0.25 to	0.11)
Conventionally measured analytes									
Glucose homeostasis									
Insulin [d]	**0.33**	(0.16 to	0.50)	**0.33**	(0.16 to	0.49)	**0.30**	(0.12 to	0.47)
C Peptide [d]	**0.31**	(0.14 to	0.47)	**0.33**	(0.17 to	0.49)	**0.31**	(0.14 to	0.47)
Fructosamine	**0.40**	(0.24 to	0.57)	**0.37**	(0.22 to	0.53)	**0.41**	(0.25 to	0.58)
HOMA2-%B [d e]	**-0.32**	(-0.49 to	-0.16)	**-0.31**	(-0.47 to	-0.14)	**-0.33**	(-0.51 to	-0.16)
HOMA2-%S [d e]	**-0.41**	(-0.58 to	-0.25)	**-0.40**	(-0.57 to	-0.24)	**-0.41**	(-0.58 to	-0.24)
HOMA2-IR [d e]	**0.41**	(0.25 to	0.58)	**0.40**	(0.24 to	0.57)	**0.41**	(0.24 to	0.58)
Liver markers									
gGT [d]	**0.33**	(0.16 to	0.49)	**0.28**	(0.12 to	0.44)	**0.24**	(0.07 to	0.41)
ALT [d]	0.06	(-0.11 to	0.23)	0.02	(-0.15 to	0.18)	-0.06	(-0.24 to	0.12)
AST [d]	0.02	(-0.15 to	0.19)	-0.03	(-0.19 to	0.13)	-0.10	(-0.27 to	0.07)
SHBG	**-0.22**	(-0.39 to	-0.05)	**-0.23**	(-0.39 to	-0.07)	-0.20	(-0.37 to	-0.02)
Adipokines									
Leptin [d]	0.00	(-0.17 to	0.17)	-0.10	(-0.25 to	0.04)	-0.09	(-0.25 to	0.06)
Adiponectin [d]	**-0.46**	(-0.62 to	-0.29)	**-0.42**	(-0.58 to	-0.26)	**-0.43**	(-0.60 to	-0.25)
Inflammatory markers									
hs-CRP [d]	0.15	(-0.02 to	0.31)	0.12	(-0.04 to	0.28)	0.13	(-0.05 to	0.30)
IL-6 [d]	**0.20**	(0.03 to	0.37)	0.16	(-0.01 to	0.32)	0.15	(-0.03 to	0.32)
tPA-antigen [d]	**0.21**	(0.04 to	0.37)	**0.20**	(0.03 to	0.36)	**0.22**	(0.04 to	0.40)
Ferritin [d]	0.07	(-0.09 to	0.24)	-0.02	(-0.19 to	0.14)	-0.03	(-0.20 to	0.14)

[a b c] False Discovery Rate (FDR) corrected *p* value <0.05 shown in bold [d] analyte log transformed [e] insulin indices missing 8 in univariate and multivariate analysis and 4 in sensitivity analysis (incalculable). GDM gestational diabetes mellitus, gGT γ-glutamyl transferase, AST aspartate aminotransferase, ALT alanine aminotransferase, SHBG sex hormone binding globulin, hs-CRP high sensitivity C-reactive protein, IL-6 interleukin-6, tPA-antigen tissue plasminogen activator antigen, HOMA2-%B steady state beta cell function, HOMA2-IR insulin resistance, HOMA2-%S insulin sensitivity.

ESM Table 4: Analyte concentrations (absolute units) at time point 1

	No GDM N=448 Mean (SD)/Median (IQR)	GDM N=198 Mean (SD)/Median (IQR)
NMR metabolites		
Total lipids		
Total lipids in chylomicrons and extremely large VLDL (umol/l)	11.7 (5.3-22.6)	17.4 (8.49-29.2)
Total lipids in very large VLDL (umol/l)	42.4 (23.3-72.7)	59.4 (35.2-97.3)
Total lipids in large VLDL (umol/l)	188 (121-300)	255 (174-375)
Total lipids in medium VLDL (mmol/l)	0.48 (0.21)	0.57 (0.24)
Total lipids in small VLDL (mmol/l)	0.56 (0.15)	0.61 (0.16)
Total lipids in very small VLDL (mmol/l)	0.51 (0.1)	0.52 (0.11)
Total lipids in IDL (mmol/l)	1.09 (0.22)	1.09 (0.22)
Total lipids in large LDL (mmol/l)	1.24 (0.27)	1.23 (0.27)
Total lipids in medium LDL (mmol/l)	0.7 (0.16)	0.69 (0.16)
Total lipids in small LDL (mmol/l)	0.46 (0.1)	0.45 (0.1)
Total lipids in very large HDL (mmol/l)	0.73 (0.19)	0.7 (0.19)
Total lipids in large HDL (mmol/l)	1.2 (0.28)	1.16 (0.29)
Total lipids in medium HDL (mmol/l)	0.99 (0.15)	1.01 (0.16)
Total lipids in small HDL (mmol/l)	1.11 (0.1)	1.14 (0.11)
Particle diameter		
Mean diameter for VLDL particles (nm)	36.3 (1)	36.7 (1.1)
Mean diameter for LDL particles (nm)	23.6 (0.1)	23.6 (0.1)
Mean diameter for HDL particles (nm)	10.3 (0.2)	10.2 (0.2)
Apolipoproteins		
Apolipoprotein A-1 (g/l)	1.76 (0.17)	1.76 (0.17)
Apolipoprotein B (g/l)	0.83 (0.14)	0.86 (0.15)
Ratio of apolipoprotein B to apolipoprotein A-1	0.48 (0.08)	0.49 (0.09)
Cholesterol		
Serum total cholesterol (mmol/l)	4.78 (0.75)	4.77 (0.76)
Total cholesterol in VLDL (mmol/l)	0.68 (0.17)	0.73 (0.18)
Total cholesterol in chylomicrons and extremely large VLDL (umol/l)	3.18 (1.68-5.08)	4.12 (2.33-6.52)
Total cholesterol in very large VLDL (umol/l)	9.14 (4.83-14.99)	12.3 (6.93-19.7)
Total cholesterol in large VLDL (umol/l)	44.1 (27.1-67.7)	57.4 (36.6-84.1)
Total cholesterol in medium VLDL (mmol/l)	0.14 (0.05)	0.16 (0.06)
Total cholesterol in small VLDL (mmol/l)	0.22 (0.05)	0.23 (0.06)
Total cholesterol in very small VLDL (mmol/l)	0.26 (0.05)	0.26 (0.05)
Remnant cholesterol (non-HDL, non-LDL) (mmol/l)	1.37 (0.29)	1.41 (0.29)
Total cholesterol in IDL (mmol/l)	0.68 (0.15)	0.68 (0.15)
Total cholesterol in LDL (mmol/l)	1.55 (0.39)	1.53 (0.39)
Total cholesterol in large LDL (mmol/l)	0.82 (0.19)	0.81 (0.19)
Total cholesterol in medium LDL (mmol/l)	0.45 (0.12)	0.45 (0.12)
Total cholesterol in small LDL (mmol/l)	0.28 (0.07)	0.27 (0.08)
Total cholesterol in HDL (mmol/l)	1.86 (0.28)	1.83 (0.29)
Total cholesterol in HDL2 (mmol/l)	1.31 (0.26)	1.27 (0.26)
Total cholesterol in HDL3 (mmol/l)	0.55 (0.03)	0.55 (0.03)
Total cholesterol in very large HDL (mmol/l)	0.34 (0.09)	0.32 (0.09)
Total cholesterol in large HDL (mmol/l)	0.58 (0.15)	0.56 (0.15)
Total cholesterol in medium HDL (mmol/l)	0.48 (0.08)	0.48 (0.09)
Total cholesterol in small HDL (mmol/l)	0.47 (0.05)	0.47 (0.05)

Cholesteryl esters		
Esterified cholesterol (mmol/l)	3.27 (0.56)	3.22 (0.56)
Cholesteryl esters in medium VLDL (mmol/l)	0.08 (0.03)	0.09 (0.03)
Cholesteryl esters in small VLDL (mmol/l)	0.14 (0.03)	0.14 (0.04)
Cholesteryl esters in very small VLDL (mmol/l)	0.17 (0.03)	0.17 (0.03)
Cholesteryl esters in IDL (mmol/l)	0.49 (0.1)	0.48 (0.1)
Cholesteryl esters in large LDL (mmol/l)	0.58 (0.15)	0.57 (0.15)
Cholesteryl esters in medium LDL (mmol/l)	0.32 (0.1)	0.31 (0.1)
Cholesteryl esters in small LDL (mmol/l)	0.2 (0.06)	0.19 (0.06)
Cholesteryl esters in very large HDL (mmol/l)	0.24 (0.06)	0.23 (0.06)
Cholesteryl esters in large HDL (mmol/l)	0.45 (0.12)	0.43 (0.11)
Cholesteryl esters in medium HDL (mmol/l)	0.38 (0.06)	0.38 (0.07)
Cholesteryl esters in small HDL (mmol/l)	0.35 (0.04)	0.35 (0.05)
Non-esterified cholesterol		
Non-esterified cholesterol (mmol/l)	1.48 (0.24)	1.48 (0.25)
Non-esterified cholesterol in chylomicrons and extremely large VLDL (umol/l)	1.17 (0.59-2.08)	1.68 (0.95-2.72)
Non-esterified cholesterol in very large VLDL (umol/l)	3.76 (2.06-6.63)	5.28 (2.94-8.74)
Non-esterified cholesterol in large VLDL (umol/l)	19.5 (11.2-31.3)	26.5 (16.9-40.2)
Non-esterified cholesterol in medium VLDL (mmol/l)	0.06 (0.03)	0.07 (0.03)
Non-esterified cholesterol in small VLDL (mmol/l)	0.08 (0.02)	0.09 (0.02)
Non-esterified cholesterol in very small VLDL (mmol/l)	0.08 (0.02)	0.08 (0.02)
Non-esterified cholesterol in IDL (mmol/l)	0.2 (0.04)	0.19 (0.05)
Non-esterified cholesterol in large LDL (mmol/l)	0.24 (0.05)	0.23 (0.05)
Non-esterified cholesterol in medium LDL (mmol/l)	0.14 (0.02)	0.14 (0.02)
Non-esterified cholesterol in small LDL (mmol/l)	0.08 (0.01)	0.08 (0.01)
Non-esterified cholesterol in very large HDL (mmol/l)	0.09 (0.03)	0.09 (0.03)
Non-esterified cholesterol in large HDL (mmol/l)	0.13 (0.04)	0.12 (0.04)
Non-esterified cholesterol in medium HDL (mmol/l)	0.1 (0.02)	0.1 (0.02)
Non-esterified cholesterol in small HDL (mmol/l)	0.12 (0.01)	0.12 (0.01)
Triacylglycerols		
Serum total triacylglycerols (mmol/l)	1.21 (0.4)	1.38 (0.45)
Triacylglycerols in VLDL (mmol/l)	0.73 (0.33)	0.87 (0.38)
Triacylglycerols in chylomicrons and extremely large VLDL (umol/l)	7.08 (2.78-14.32)	10.6 (4.8-18.7)
Triacylglycerols in very large VLDL (umol/l)	26.4 (14.4-45.8)	37.2 (23-61)
Triacylglycerols in large VLDL (umol/l)	108 (72-176)	147 (101-224)
Triacylglycerols in medium VLDL (mmol/l)	0.24 (0.12)	0.29 (0.14)
Triacylglycerols in small VLDL (mmol/l)	0.2 (0.07)	0.23 (0.08)
Triacylglycerols in very small VLDL (mmol/l)	0.11 (0.03)	0.12 (0.03)
Triacylglycerols in IDL (mmol/l)	0.12 (0.03)	0.13 (0.03)
Triacylglycerols in LDL (mmol/l)	0.2 (0.05)	0.21 (0.05)
Triacylglycerols in large LDL (mmol/l)	0.11 (0.03)	0.12 (0.03)
Triacylglycerols in medium LDL (mmol/l)	0.05 (0.01)	0.06 (0.01)
Triacylglycerols in small LDL (mmol/l)	0.03 (0.01)	0.03 (0.01)
Triacylglycerols in HDL (mmol/l)	0.16 (0.03)	0.18 (0.03)
Triacylglycerols in very large HDL (mmol/l)	0.02 (0.01)	0.03 (0.01)
Triacylglycerols in large HDL (mmol/l)	0.05 (0.02)	0.05 (0.02)
Triacylglycerols in medium HDL (mmol/l)	0.04 (0.01)	0.05 (0.01)
Triacylglycerols in small HDL (mmol/l)	0.05 (0.01)	0.05 (0.01)
Phospholipids		
Total phospholipids (mmol/l)	3.37 (0.37)	3.40 (0.38)
Phospholipids in VLDL (mmol/l)	0.43 (0.21)	0.47 (0.13)
Phospholipids in chylomicrons and extremely large VLDL (umol/l)	1.51 (0.67-2.95)	2.35 (1.15-3.81)

Phospholipids in very large VLDL (umol/l)	6.66 (3.71-11.84)	9.61 (5.32-15.75)
Phospholipids in large VLDL (umol/l)	35 (22.3-55.4)	45.7 (31.3-67.6)
Phospholipids in medium VLDL (mmol/l)	0.1 (0.04)	0.11 (0.05)
Phospholipids in small VLDL (mmol/l)	0.13 (0.03)	0.14 (0.04)
Phospholipids in very small VLDL (mmol/l)	0.15 (0.03)	0.15 (0.03)
Phospholipids in IDL (mmol/l)	0.29 (0.06)	0.28 (0.06)
Phospholipids in LDL (mmol/l)	0.64 (0.10)	0.65 (0.10)
Phospholipids in large LDL (mmol/l)	0.31 (0.05)	0.31 (0.05)
Phospholipids in medium LDL (mmol/l)	0.19 (0.03)	0.19 (0.03)
Phospholipids in small LDL (mmol/l)	0.14 (0.02)	0.14 (0.02)
Phospholipids in HDL (mmol/l)	2.01 (0.28)	2.0 (0.28)
Phospholipids in very large HDL (mmol/l)	0.37 (0.1)	0.35 (0.1)
Phospholipids in large HDL (mmol/l)	0.57 (0.12)	0.55 (0.13)
Phospholipids in medium HDL (mmol/l)	0.47 (0.07)	0.48 (0.07)
Phospholipids in small HDL (mmol/l)	0.6 (0.07)	0.62 (0.08)
Total phosphoglycerides (mmol/l)	2.56 (0.31)	2.58 (0.29)
Ratio of triacylglycerols to phosphoglycerides (mmol/l)	0.56 (0.15)	0.62 (0.17)
Phosphatidylcholine and other cholines (mmol/l)	2.33 (0.3)	2.33 (0.29)
Sphingomyelins (mmol/l)	0.46 (0.07)	0.45 (0.08)
Total cholines (mmol/l)	2.92 (0.33)	2.92 (0.33)
Fatty acids		
Total fatty acids (mmol/l)	13.7 (1.9)	14.2 (2)
Cholesteryl esters in chylomicrons and extremely large VLDL (umol/l)	1.9 (1.03-3.05)	2.43 (1.45-3.84)
Cholesteryl esters in very large VLDL (umol/l)	5.37 (2.83-8.31)	7.01 (4.01-10.72)
Cholesteryl esters in large VLDL (umol/l)	24.4 (15.7-36)	30.6 (20.2-43.9)
Estimated degree of unsaturation	1.13 (0.04)	1.12 (0.05)
22:6, docosahexaenoic acid (mmol/l)	0.23 (0.04)	0.24 (0.05)
18:2, linoleic acid (mmol/l)	3.55 (0.55)	3.56 (0.57)
Omega-3 fatty acids (mmol/l)	0.67 (0.13)	0.69 (0.14)
Omega-6 fatty acids (mmol/l)	4.18 (0.57)	4.21 (0.59)
Polyunsaturated fatty acids (mmol/l)	4.85 (0.66)	4.9 (0.7)
Monounsaturated fatty acids; 16:1, 18:1 (mmol/l)	3.76 (0.68)	3.97 (0.74)
Saturated fatty acids (mmol/l)	5.1 (0.74)	5.28 (0.78)
Ratio of 22:6 docosahexaenoic acid to total fatty acids (%)	1.71 (0.25)	1.7 (0.29)
Ratio of 18:2 linoleic acid to total fatty acids (%)	26 (2.5)	25.2 (2.5)
Ratio of omega-3 fatty acids to total fatty acids (%)	4.87 (0.7)	4.85 (0.79)
Ratio of omega-6 fatty acids to total fatty acids (%)	30.6 (2.3)	29.9 (2.4)
Ratio of polyunsaturated fatty acids to total fatty acids (%)	35.5 (2.6)	34.7 (2.8)
Ratio of monounsaturated fatty acids to total fatty acids (%)	27.3 (2)	27.9 (2)
Ratio of saturated fatty acids to total fatty acids (%)	37.2 (1.3)	37.3 (1.3)
Glycolysis related		
Glucose (mmol/l)	3.72 (0.53)	4 (0.78)
Lactate (mmol/l)	1.3 (0.53)	1.33 (0.44)
Pyruvate (umol/l)	95.1 (32.3)	105 (46)
Citrate (umol/l)	107 (17)	111 (17)
Amino acids		
Alanine (umol/l)	374 (40)	380 (41)
Glutamine (umol/l)	375 (46)	364 (46)
Glycine (umol/l)	219 (24)	214 (21)
Branched chain amino acids		
Isoleucine (umol/l)	45.5 (12.6)	50.1 (13.5)
Leucine (umol/l)	62 (13.9)	67.1 (14.6)
Valine (umol/l)	131 (31)	140 (30)

Aromatic amino acids		
Phenylalanine (umol/l)	74.4 (10)	77 (10.2)
Tyrosine (umol/l)	40.8 (8.8)	42.2 (8.2)
Histidine (umol/l)	67.3 (7.2)	66.6 (6.3)
Inflammatory marker (NMR)		
Glycoprotein acetlys, mainly a1-acid glycoprotein (mmol/l)	1.46 (0.12)	1.52 (0.15)
Ketone bodies		
Acetoacetate (umol/l)	21.2 (16.4-27.3)	23.7 (17.7-31.4)
3-hydroxybutyrate (umol/l)	87.7 (73.1-113.2)	95.3 (74.6-122.8)
Other (NMR)		
Acetate (umol/l)	38.6 (34.8-43.4)	39.1 (35.8-43.3)
Creatinine (umol/l)	0.04 (0.01)	0.04 (0.01)
Albumin (signal area)	0.08 (0)	0.08 (0)
Conventionally measured analytes		
Glucose homeostasis		
Insulin (pmol/l)	156 (84.7-128)	226 (130-504)
C-peptide (nmol/l)	1.25 (0.84-1.99)	1.52 (1.06-2.39)
HbA_{1c} (%)	4.8 (0.33)	5 (0.38)
HbA_{1c} (mmol/mol)	29.1 (3.6)	31.3 (4.2)
Fructosamine (umol/l)	188 (19)	195 (22)
Liver markers		
gGT (U/L)	12 (8-18)	15 (10-25)
ALT (U/L)	16.4 (12.2-23.6)	17.1 (12.9-25.1)
AST (U/L)	22.3 (17.8-27.6)	23.7 (18.7-29.4)
SHBG (nmol/l)	436 (129)	387 (115)
hs-CRP (nmol/l)	62.4 (40.2-104)	66.3 (47.7-111)
Adipokines		
Leptin (pg/ml)	65.9 (46.9-85.2)	65.9 (47.7-88.2)
Adiponectin (ug/ml)	10.3 (7.2-16)	7.57 (4.64-12.33)
Inflammatory markers		
Ferritin (pmol/l)	105 (59.8-180)	119 (76.6-193)
IL-6 (pg/ml)	2.94 (2.04-4.56)	3 (1.98-4.47)
tPA-antigen (ng/ml)	6.75 (5.3-8.61)	7.35 (5.7-9.74)
Miscellaneous		
Vitamin D (nmol/l)	40.0 (25.8-58.8)	37.0 (22.8-51.5)
hPL (z score)	0 (1)	-0.2 (0.9)

GDM gestational diabetes mellitus, gGT γ-glutamyl transferase, AST aspartate aminotransferase, ALT alanine aminotransferase, SHBG sex hormone binding globulin, hs-CRP high sensitivity C-reactive protein, IL-6 interleukin-6, tPA-antigen tissue plasminogen activator antigen, hPL human placental lactogen

ESM Table 5: Analyte concentrations (absolute units) at time point 2

	No GDM N=498 Mean (SD)/Median (IQR)	GDM N=198 Mean(SD)/Median (IQR)
NMR metabolites		
Total lipids		
Total lipids in chylomicrons and extremely large VLDL (umol/l)	20.3 (10.4-30.3)	22.9 (11.9-43.9)
Total lipids in very large VLDL (umol/l)	70.2 (43.6-102.7)	80.8 (49.6-145.5)
Total lipids in large VLDL (umol/l)	297 (200-419)	337 (228-564)
Total lipids in medium VLDL (mmol/l)	0.65 (0.26)	0.74 (0.33)
Total lipids in small VLDL (mmol/l)	0.7 (0.2)	0.75 (0.22)
Total lipids in very small VLDL (mmol/l)	0.62 (0.14)	0.62 (0.15)
Total lipids in IDL (mmol/l)	1.3 (0.3)	1.26 (0.3)
Total lipids in large LDL (mmol/l)	1.48 (0.36)	1.42 (0.37)
Total lipids in medium LDL (mmol/l)	0.84 (0.22)	0.8 (0.22)
Total lipids in small LDL (mmol/l)	0.54 (0.13)	0.52 (0.13)
Total lipids in very large HDL (mmol/l)	0.82 (0.2)	0.78 (0.21)
Total lipids in large HDL (mmol/l)	1.26 (0.29)	1.2 (0.3)
Total lipids in medium HDL (mmol/l)	0.96 (0.15)	0.96 (0.16)
Total lipids in small HDL (mmol/l)	1.11 (0.1)	1.12 (0.11)
Particle diameter		
Mean diameter for VLDL particles (nm)	36.7 (1)	37 (1.2)
Mean diameter for LDL particles (nm)	23.6 (0.1)	23.6 (0.1)
Mean diameter for HDL particles (nm)	10.3 (0.2)	10.3 (0.2)
Apolipoproteins		
Apolipoprotein A-1 (g/l)	1.83 (0.18)	1.8 (0.17)
Apolipoprotein B (g/l)	0.99 (0.2)	0.99 (0.2)
Ratio of apolipoprotein B to apolipoprotein A-1	0.54 (0.11)	0.55 (0.12)
Cholesterol		
Total cholesterol in chylomicrons and extremely large VLDL (umol/l)	5.34 (3.45-7.4)	5.92 (3.72-9.48)
Total cholesterol in very large VLDL (umol/l)	15 (9.6-21.1)	16.5 (10.9-27.9)
Total cholesterol in large VLDL (umol/l)	69.5 (46.8-97.3)	79 (53-123.9)
Serum total cholesterol (mmol/l)	5.43 (0.96)	5.27 (0.96)
Total cholesterol in VLDL (mmol/l)	0.86 (0.23)	0.91 (0.25)
Total cholesterol in medium VLDL (mmol/l)	0.19 (0.07)	0.21 (0.08)
Total cholesterol in small VLDL (mmol/l)	0.27 (0.07)	0.28 (0.08)
Total cholesterol in very small VLDL (mmol/l)	0.3 (0.07)	0.3 (0.07)
Remnant cholesterol (non-HDL, non-LDL) (mmol/l)	1.67 (0.39)	1.68 (0.4)
Total cholesterol in IDL (mmol/l)	0.8 (0.2)	0.77 (0.2)
Total cholesterol in LDL (mmol/l)	1.86 (0.53)	1.76 (0.53)
Total cholesterol in large LDL (mmol/l)	0.97 (0.26)	0.93 (0.27)
Total cholesterol in medium LDL (mmol/l)	0.55 (0.16)	0.52 (0.17)
Total cholesterol in small LDL (mmol/l)	0.34 (0.1)	0.32 (0.1)
Total cholesterol in HDL (mmol/l)	1.91 (0.29)	1.84 (0.3)

Total cholesterol in HDL2 (mmol/l)	1.33 (0.27)	1.26 (0.28)
Total cholesterol in HDL3 (mmol/l)	0.58 (0.03)	0.57 (0.03)
Total cholesterol in very large HDL (mmol/l)	0.37 (0.09)	0.36 (0.1)
Total cholesterol in large HDL (mmol/l)	0.61 (0.15)	0.58 (0.16)
Total cholesterol in medium HDL (mmol/l)	0.45 (0.08)	0.44 (0.09)
Total cholesterol in small HDL (mmol/l)	0.47 (0.05)	0.46 (0.05)
Cholesteryl esters		
Esterified cholesterol (mmol/l)	3.42 (0.68)	3.3 (0.68)
Cholesteryl esters in chylomicrons and extremely large VLDL (umol/l)	3.36 (2.17-4.52)	3.63 (2.37-5.36)
Cholesteryl esters in very large VLDL (umol/l)	8.25 (5.45-11.74)	9.34 (6.23-15.09)
Cholesteryl esters in large VLDL (umol/l)	36.5 (25.3-50.4)	40.7 (28.2-61)
Cholesteryl esters in medium VLDL (mmol/l)	0.11 (0.04)	0.12 (0.04)
Cholesteryl esters in small VLDL (mmol/l)	0.17 (0.05)	0.17 (0.05)
Cholesteryl esters in very small VLDL (mmol/l)	0.2 (0.04)	0.2 (0.05)
Cholesteryl esters in IDL (mmol/l)	0.57 (0.14)	0.56 (0.14)
Cholesteryl esters in large LDL (mmol/l)	0.7 (0.2)	0.67 (0.2)
Cholesteryl esters in medium LDL (mmol/l)	0.39 (0.14)	0.37 (0.14)
Cholesteryl esters in small LDL (mmo/l)	0.24 (0.08)	0.22 (0.09)
Cholesteryl esters in very large HDL (mmol/l)	0.27 (0.07)	0.26 (0.07)
Cholesteryl esters in large HDL (mmol/l)	0.47 (0.12)	0.45 (0.12)
Cholesteryl esters in medium HDL (mmol/l)	0.36 (0.07)	0.35 (0.07)
Cholesteryl esters in small HDL (mmol/l)	0.36 (0.05)	0.35 (0.05)
Non-esterified cholesterol		
Non-esterified cholesterol (mmol/l)	1.48 (0.28)	1.44 (0.28)
Non-esterified cholesterol in chylomicrons and extremely large VLDL (umol/l)	2.03 (1.19-2.99)	2.36 (1.38-4.37)
Non-esterified cholesterol in very large VLDL (umol/l)	6.72 (4.14-9.55)	7.49 (4.55-12.97)
Non-esterified cholesterol in large VLDL (umol/l)	32.8 (21.3-46.8)	37.2 (25.2-64.4)
Non-esterified cholesterol in medium VLDL (mmol/l)	0.08 (0.03)	0.09 (0.04)
Non-esterified cholesterol in small VLDL (mmol/l)	0.11 (0.03)	0.11 (0.03)
Non-esterified cholesterol in very small VLDL (mmol/l)	0.1 (0.02)	0.1 (0.03)
Non-esterified cholesterol in IDL (mmol/l)	0.23 (0.06)	0.22 (0.06)
Non-esterified cholesterol in large LDL (mmol/l)	0.28 (0.07)	0.26 (0.07)
Non-esterified cholesterol in medium LDL (mmol/l)	0.16 (0.03)	0.15 (0.03)
Non-esterified cholesterol in small LDL (mmol/l)	0.09 (0.02)	0.09 (0.02)
Non-esterified cholesterol in very large HDL (mmol/l)	0.11 (0.03)	0.1 (0.03)
Non-esterified cholesterol in large HDL (mmol/l)	0.14 (0.04)	0.13 (0.04)
Non-esterified cholesterol in medium HDL (mmol/l)	0.09 (0.02)	0.09 (0.02)
Non-esterified cholesterol in small HDL (mmol/l)	0.11 (0.01)	0.12 (0.01)
Triacylglycerols		
Serum total triacylglycerols (mmol/l)	1.6 (0.51)	1.78 (0.64)
Triacylglycerols in VLDL (mmol/l)	0.98 (0.4)	1.14 (0.53)
Triacylglycerols in chylomicrons and extremely large VLDL (umol/l)	11.7 (5.8-18.8)	13.4 (6.51-28.6)
Triacylglycerols in very large VLDL (umol/l)	42.8 (26.6-64.8)	49.4 (30.1-89.9)
Triacylglycerols in large VLDL (umol/l)	171 (116-244)	195 (131-327)

Triacylglycerols in medium VLDL (mmol/l)	0.33 (0.14)	0.38 (0.19)
Triacylglycerols in small VLDL (mmol/l)	0.27 (0.09)	0.29 (0.11)
Triacylglycerols in very small VLDL (mmol/l)	0.14 (0.04)	0.15 (0.04)
Triacylglycerols in IDL (mmol/l)	0.16 (0.04)	0.17 (0.04)
Triacylglycerols in LDL (mmol/l)	0.26 (0.07)	0.27 (0.07)
Triacylglycerols in large LDL (mmol/l)	0.15 (0.04)	0.15 (0.04)
Triacylglycerols in medium LDL (mmol/l)	0.07 (0.02)	0.07 (0.02)
Triacylglycerols in small LDL (mmol/l)	0.04 (0.01)	0.04 (0.01)
Triacylglycerols in HDL (mmol/l)	0.2 (0.04)	0.21 (0.04)
Triacylglycerols in very large HDL (mmol/l)	0.03 (0.01)	0.03 (0.01)
Triacylglycerols in large HDL (mmol/l)	0.06 (0.02)	0.06 (0.02)
Triacylglycerols in medium HDL (mmol/l)	0.05 (0.01)	0.05 (0.01)
Triacylglycerols in small HDL (mmol/l)	0.06 (0.01)	0.06 (0.02)
Phospholipids		
Total phospholipids (mmol/l)	3.67 (0.42)	3.65 (0.41)
Phospholipids in VLDL (mmol/l)	0.55 (0.16)	0.60 (0.19)
Phospholipids in chylomicrons and extremely large VLDL (umol/l)	2.78 (1.47-4.17)	3.23 (1.68-5.98)
Phospholipids in very large VLDL (umol/l)	11.9 (7.2-17.1)	13.9 (8.07-24.5)
Phospholipids in large VLDL (umol/l)	56.1 (38.4-78.3)	64.1 (43.6-106.8)
Phospholipids in medium VLDL (mmol/l)	0.13 (0.05)	0.15 (0.07)
Phospholipids in small VLDL (mmol/l)	0.16 (0.04)	0.17 (0.05)
Phospholipids in very small VLDL (mmol/l)	0.18 (0.05)	0.18 (0.05)
Phospholipids in IDL (mmol/l)	0.33 (0.07)	0.32 (0.08)
Phospholipids in LDL (mmol/l)	0.74 (0.14)	0.72 (0.14)
Phospholipids in large LDL (mmol/l)	0.36 (0.07)	0.35 (0.07)
Phospholipids in medium LDL (mmol/l)	0.22 (0.04)	0.22 (0.04)
Phospholipids in small LDL (mmol/l)	0.16 (0.03)	0.16 (0.03)
Phospholipids in HDL (mmol/l)	2.04 (0.28)	2.01 (0.29)
Phospholipids in very large HDL (mmol/l)	0.41 (0.11)	0.39 (0.11)
Phospholipids in large HDL (mmol/l)	0.59 (0.12)	0.56 (0.13)
Phospholipids in medium HDL (mmol/l)	0.46 (0.07)	0.47 (0.07)
Phospholipids in small HDL (mmol/l)	0.58 (0.08)	0.59 (0.09)
Total phosphoglycerides (mmol/l)	2.57 (0.33)	2.56 (0.34)
Ratio of triacylglycerols to phosphoglycerides	0.65 (0.15)	0.71 (0.19)
Phosphatidylcholine and other cholines (mmol/l)	2.36 (0.34)	2.34 (0.33)
Sphingomyelins (mmol/l)	0.42 (0.08)	0.41 (0.08)
Total cholines (mmol/l)	2.81 (0.35)	2.78 (0.36)
Fatty acids		
Total fatty acids (mmol/l)	14 (2.1)	14.2 (2.3)
Estimated degree of unsaturation	1.1 (0.04)	1.09 (0.05)
22:6, docosahexaenoic acid (mmol/l)	0.21 (0.04)	0.22 (0.04)
18:2, linoleic acid (mmol/l)	3.52 (0.62)	3.46 (0.63)
Omega-3 fatty acids (mmol/l)	0.64 (0.12)	0.65 (0.12)
Omega-6 fatty acids (mmol/l)	4.07 (0.67)	4.02 (0.68)
Polyunsaturated fatty acids (mmol/l)	4.71 (0.76)	4.67 (0.77)

Monounsaturated fatty acids; 16:1, 18:1 (mmol/l)	4.15 (0.7)	4.3 (0.8)
Saturated fatty acids (mmol/l)	5.09 (0.8)	5.23 (0.88)
Ratio of 22:6 docosahexaenoic acid to total fatty acids (%)	1.55 (0.23)	1.54 (0.26)
Ratio of 18:2 linoleic acid to total fatty acids (%)	25.2 (2.1)	24.4 (2.3)
Ratio of omega-3 fatty acids to total fatty acids (%)	4.59 (0.67)	4.58 (0.71)
Ratio of omega-6 fatty acids to total fatty acids (%)	29.2 (2)	28.4 (2.3)
Ratio of polyunsaturated fatty acids to total fatty acids (%)	33.8 (2.3)	33 (2.7)
Ratio of monounsaturated fatty acids to total fatty acids (%)	29.7 (1.5)	30.2 (1.7)
Ratio of saturated fatty acids to total fatty acids (%)	36.5 (1.2)	36.8 (1.4)
Glycolysis related		
Glucose (mmol/l)	3.66 (0.66)	4.18 (0.97)
Lactate (mmol/l)	1.39 (0.49)	1.42 (0.39)
Pyruvate (umol/l)	90.3 (34)	106 (35)
Citrate (umol/l)	113 (16)	119 (16)
Amino acids		
Alanine (umol/l)	373 (39)	382 (39)
Glutamine (umol/l)	370 (41)	362 (40)
Glycine (umol/l)	219 (26)	221 (23)
Branched chain amino acids		
Isoleucine (umol/l)	45 (9.1)	49.5 (10.7)
Leucine (umol/l)	58.6 (9.3)	63.4 (10.4)
Valine (umol/l)	117 (19)	125 (20)
Aromatic amino acids		
Phenylalanine (umol/l)	76.5 (9.7)	80.3 (10.1)
Tyrosine (umol/l)	36.2 (5.6)	37.6 (6)
Histidine (umol/l)	65.4 (6.2)	65.8 (6)
Ketone bodies		
Acetoacetate (umol/l)	24 (18.5-29.7)	27 (21.1-33)
3-hydroxybutyrate (umol/l)	114 (94-138)	120 (101-145)
Other (NMR)		
Creatinine (mmol/l)	0.04 (0.01)	0.04 (0.01)
Albumin (signal area)	0.08 (0)	0.08 (0)
Acetate (umol/l)	42.2 (37.6-49.2)	41.4 (37.5-48.8)
Inflammatory Marker (NMR)		
Glycoprotein acetlys, mainly a1-acid glycoprotein (mmol/l)	1.55 (0.14)	1.61 (0.15)
Conventionally measured analytes		
Glucose homeostasis		
Fructosamine (umol/l)	178 (13)	184 (16)
Insulin (pmol/l)	113 (81.3-165)	151 (108-203)
C Peptide (nmol/l)	1.04 (0.82-1.37)	1.30 (0.99-1.61)
Liver markers		
gGT (U/L)	12 (8-19)	14 (10-24)
ALT (U/L)	14.1 (10.6-18.8)	15 (10.7-20.2)
AST (U/L)	21.3 (17.2-25.6)	21.7 (17.8-26.5)
SHBG (nmol/l)	512 (149)	481 (133)

Adipokines		
Leptin (pg/ml)	65.8 (48.8-86.6)	62.9 (47.3-89.6)
Adiponectin (ug/ml)	10.2 (7.1-14.5)	7.92 (5.12-11.39)
Inflammatory markers		
hs-CRP (nmol/l)	56.7 (36.9-88.1)	64.2 (41.1-96.4)
Ferritin (pmol/l)	38.6 (26.3-68.5)	43.6 (28.3-73.7)
IL-6 (pg/ml)	1.92 (1.45-2.46)	2.08 (1.67-2.63)
tPA antigen (ng/ml)	7.19 (5.49-10.03)	8.26 (6.02-11.89)
Insulin indices [a]		
HOMA2-%B	214 (170-277)	195 (156-227)
HOMA2-%S	44.2 (30.8-60.5)	31.8 (24-43.3)
HOMA2-IR	2.3 (1.7-3.2)	3.15 (2.3-4.2)

GDM gestational diabetes mellitus, gGT γ-glutamyl transferase, AST aspartate aminotransferase, ALT alanine aminotransferase, SHBG sex hormone binding globulin, hs-CRP high sensitivity C-reactive protein, IL-6 interleukin-6, tPA-antigen tissue plasminogen activator antigen, HOMA2-%B steady state beta cell function, HOMA2-IR insulin resistance, HOMA2-%S insulin sensitivity. [a] Missing insulin indices as incalculable: 8
